# Acute myeloid leukemia immunopeptidome reveals HLA presentation of mutated nucleophosmin

**DOI:** 10.1371/journal.pone.0219547

**Published:** 2019-07-10

**Authors:** Rupa Narayan, Niclas Olsson, Lisa E. Wagar, Bruno C. Medeiros, Everett Meyer, Debra Czerwinski, Michael S. Khodadoust, Lichao Zhang, Liora Schultz, Mark M. Davis, Joshua E. Elias, Ron Levy

**Affiliations:** 1 Department of Medicine, Division of Hematology, Stanford University, Stanford, CA, United States of America; 2 Department of Chemical and Systems Biology, Stanford University, Stanford, CA, United States of America; 3 Department of Microbiology & Immunology, Stanford University, Stanford, CA, United States of America; 4 Department of Medicine, Division of Blood and Marrow Transplantation, Stanford University, Stanford, CA, United States of America; 5 Department of Medicine, Division of Oncology, Stanford University, Stanford, CA, United States of America; 6 Department of Pediatrics, Division of Hematology/Oncology, Stanford University, Stanford, CA, United States of America; 7 Howard Hughes Medical Institute, Stanford University, Stanford, CA, United States of America; University of Texas MD Anderson Cancer Center, UNITED STATES

## Abstract

Somatic mutations in cancer are a potential source of cancer specific neoantigens. Acute myeloid leukemia (AML) has common recurrent mutations shared between patients in addition to private mutations specific to individuals. We hypothesized that neoantigens derived from recurrent shared mutations would be attractive targets for future immunotherapeutic approaches. Here we sought to study the HLA Class I and II immunopeptidome of thirteen primary AML tumor samples and two AML cell lines (OCI-AML3 and MV4-11) using mass spectrometry to evaluate for endogenous mutation-bearing HLA ligands from common shared AML mutations. We identified two endogenous, mutation-bearing HLA Class I ligands from nucleophosmin (NPM1). The ligands, AVEEVSLRK from two patient samples and C(cys)LAVEEVSL from OCI-AML3, are predicted to bind the common HLA haplotypes, HLA-A*03:01 and HLA-A*02:01 respectively. Since NPM1 is mutated in approximately one-third of patients with AML, the finding of endogenous HLA ligands from mutated NPM1 supports future studies evaluating immunotherapeutic approaches against this shared target, for this subset of patients with AML.

## Introduction

The major cause of therapeutic failure in AML is disease relapse [1]. Novel approaches are needed to target AML in a durable and specific manner. Immunotherapy, using the cytolytic capacity of the adaptive immune system for specific anti-tumor targeting, is one such potential approach. While several leukemia-associated antigens (LAA) have been identified (such as WT1, Cyclin A1) [2,3], leukemia specific antigens (LSA) have not been as well defined.

We hypothesized that somatic mutations in AML may potentially result in novel antigens (neoantigens). Neoantigens have been predicted in other tumors by applying *in silico* human leukocyte antigen (HLA) binding algorithms to mutations identified through exome sequencing [4–6]. Alternatively, class I and II HLA immunopeptidome analyses using mass spectrometry (MS) combined with exome sequencing of primary tumor samples have identified endogenous neoantigens in melanoma and lymphoma [7–9]. Although AML has a low mutation burden [10,11], and therefore relatively few predicted neoantigens, recurrent ‘hotspot mutations’ are shared by substantial numbers of patients [11,12]. Such hotspot mutations are often clonal driver mutations [11,13], and therefore may be more effective targets than neoantigens derived from sub-clonal and/or passenger mutations. We hypothesized that shared HLA ligands corresponding to recurrent shared mutations exist, which if identified, could potentially lead to future development of novel immunotherapy for substantial numbers of patients.

We searched for such shared HLA ligands by predicting *in silico* HLA Class I binding affinities of common recurrent AML mutations and by directly surveying the HLA Class I and II immunopeptidomes of thirteen primary AML tumor samples and two AML cell lines, OCI-AML3 and MV4-11, using mass spectrometry (MS). While one previous study reported the HLA Class I and II immunopeptidome of primary AML tumor samples evaluating non-mutated leukemia associated HLA ligands [14], we focused our detection efforts on identifying mutant HLA ligands from tumor samples known to bear common recurrent mutations. Our investigation revealed the endogenous Class I presentation of a known recurrent mutation involving nucleophosmin (NPM1). *NPM1* mutations in adult AML generally arise from base pair insertions, which create frameshifts and consequently, novel C terminus sequences [15]. The frameshift nature of this mutation produces multiple candidate HLA ligands. Here, we identified HLA Class I ligands which spanned the mutated C terminal sequences, including AVEEVSLRK from two primary patient tumor samples and C(cys)LAVEEVSL from OCI-AML3. These peptides are predicted to bind two common HLA haplotypes, HLA-A*03:01 and HLA-A*02:01, respectively. Since NPM1 is recurrently mutated in 27–35% of adult AML [11,12], our finding of endogenously presented HLA ligands from this recurrent, shared mutation in the context of common HLA haplotypes may have future immunotherapeutic applications.

## Materials and methods

### Analysis of predicted HLA ligands from common recurrent AML mutations

We used NetMHC3.4 [16,17] to predict HLA Class I binding affinities of 9-11mer peptides overlapping the mutated region of common recurrent AML mutations (NPM1 mutation A/D, FLT3-TKD (D835Y, D835E, D835H), IDH1 (R132C, R132H), IDH2 (R140Q, R172K), KIT (D816V, D816Y, Y418S), RAS (G12C, G12D, G12V, G13D, Q61H, Q61K, Q61P, Q61R), DNMT3A (R882H, R882C)) to available HLA-A, B, and C alleles, and compared these values to those predicted from the corresponding wildtype peptide sequences. Peptides with predicted affinity of <500 nM (half maximum inhibitory concentration, IC50) were considered as predicted ligands, and those with predicted affinities <100 nM as strong binders. The number of predicted ligands versus HLA alleles was plotted using GraphPad Prism software (La Jolla, CA).

### Chart review and samples

Peripheral blood (PB) and leukapheresis (LP) primary AML tumor samples were collected in the Stanford Hematology tissue bank with informed consent in accordance with the Declaration of Helsinki. IRB approval (#28969, #32256) was obtained for review of medical charts and evaluation of stored tumor samples. Mutational data (S1 Table) and HLA type (Stanford Blood Center using sanger sequencing) from patient samples, previously performed as part of clinical care, were annotated from medical records. Known *FLT3-ITD* and *NPM1* mutations from patient samples were confirmed with sanger sequencing (see S1 File for supplementary methods). Peripheral blood mononuclear cells (PBMCs) were isolated from patient tumor samples using Ficoll-Paque density gradient centrifugation and placed in 20% fetal calf serum with 10% DMSO, with storage in either -80°C or vapor phase of liquid nitrogen until use.

The OCI-AML3 cell line, which has NPM1 mutation A (p.W88fs*12) [18], was a kind gift of Dr. Beverly Mitchell. The MV4-11 cell line, which has mutated FLT3-ITD [19], was obtained from ATCC. The two cell lines were grown to 2 X 10^9^ in complete RPM1 (10% FBS) and complete IMDM (10% FBS) respectively. Cells were washed twice in PBS, flash frozen in liquid nitrogen, and stored in -80°C until use. Kashi clinical labs (Portland, Oregon) was used to obtain the HLA-A, B, C and HLA-DR typing of both cell lines and to confirm typing of one patient tumor sample (AML003). HLA-ABC and HLA-DR expression in primary AML tumor samples and AML cell lines was analyzed using flow cytometry (see S1 File).

### MHC-Class I and II immunopeptidome analysis by mass spectrometry

MHC-Class I and II immunopeptidomes were measured in parallel from primary AML tumor samples (1 X 10^8^ cells per MHC preparation) and AML cell lines (1 X 10^9^ cells per MHC preparation) as previously described (see S1 File) [8,20,21]. Isolated HLA peptides were reconstituted in 12 μl of 0.1% formic acid and analyzed on an LTQ Orbitrap Elite mass spectrometer (Thermo Fisher Scientific, Bremen, Germany) or a Fusion Lumos mass spectrometer (Thermo Fisher Scientific, San Jose, USA). Peptides were separated by capillary reverse phase chromatography on 20–24 cm reversed phase columns (100 μm inner diameter, packed in-house with ReproSil-Pur C18-AQ 3.0 m resin (Dr. Maisch GmbH)) using two-step linear gradients with increasing acetonitrile as previously described (see S1 File) [8,21]. All primary AML tumor samples were measured with the Orbitrap Elite mass spectrometer and analyzed three times with complementary acquisition methods. The two cell line specimens (OCI-AML3 and MV4-11) were analyzed with the Fusion Lumos tribrid mass spectrometer.

### Computational identification of immunopeptidomes from mass spectra

All tandem mass spectra were queried against a personalized “target-decoy” protein sequence database [22], using both SEQUEST and PEAKS DB search engines (PEAKS Studio 8, Bioinformatics Solutions Inc.) [23]. This database consisted of the human proteome (UniProtKB, version February 2016) along with sequences from recurrent AML mutations (S1 Table). Decoy entries were generated by protein sequence reversal and appended to unaltered “target” sequences. To improve high-confidence peptide identification, the spectra were also interpreted by *de novo* sequencing (PEAKS Studio 8). For all searches, the parent mass error tolerance was set to 10 ppm and the fragment mass error tolerance to 0.02 Da. For SEQUEST and PEAKS DB, enzyme specificity was set to none and oxidation of methionines and deamidation (N,Q), cysteinylation, and phosphorylation (S, T, Y) were considered as variable modifications. High-confidence peptide identifications were selected at a 1% false discovery rate (FDR) with a modified version of the Percolator algorithm [24], unless otherwise indicated. Peptide data have been deposited in the PRIDE Archive [25] at www.ebi.ac.uk/pride/archive (accession #PXD012083). Post-translational modifications were counted as distinct. The extents to which peptides and their source proteins differed between all patient tumor samples was measured as previously described [8]. Enriched gene ontologies were assessed from source genes of the peptides we identified using GOrilla (Gene Ontology Enrichment Analysis and Visualization Tool) [26,27]. Peptides identical to empirically identified mutation-bearing HLA Class I peptides were synthesized by Elim Biopharmaceuticals (Hayward, CA) with a purity of >90%. These were dissolved in 0.1% formic acid and analyzed by MS to allow comparison of spectra between the synthetic and endogenously identified peptides.

## Results

### Putative peptides from common recurrent AML mutations are predicted HLA Class I ligands

**Fig 1:**
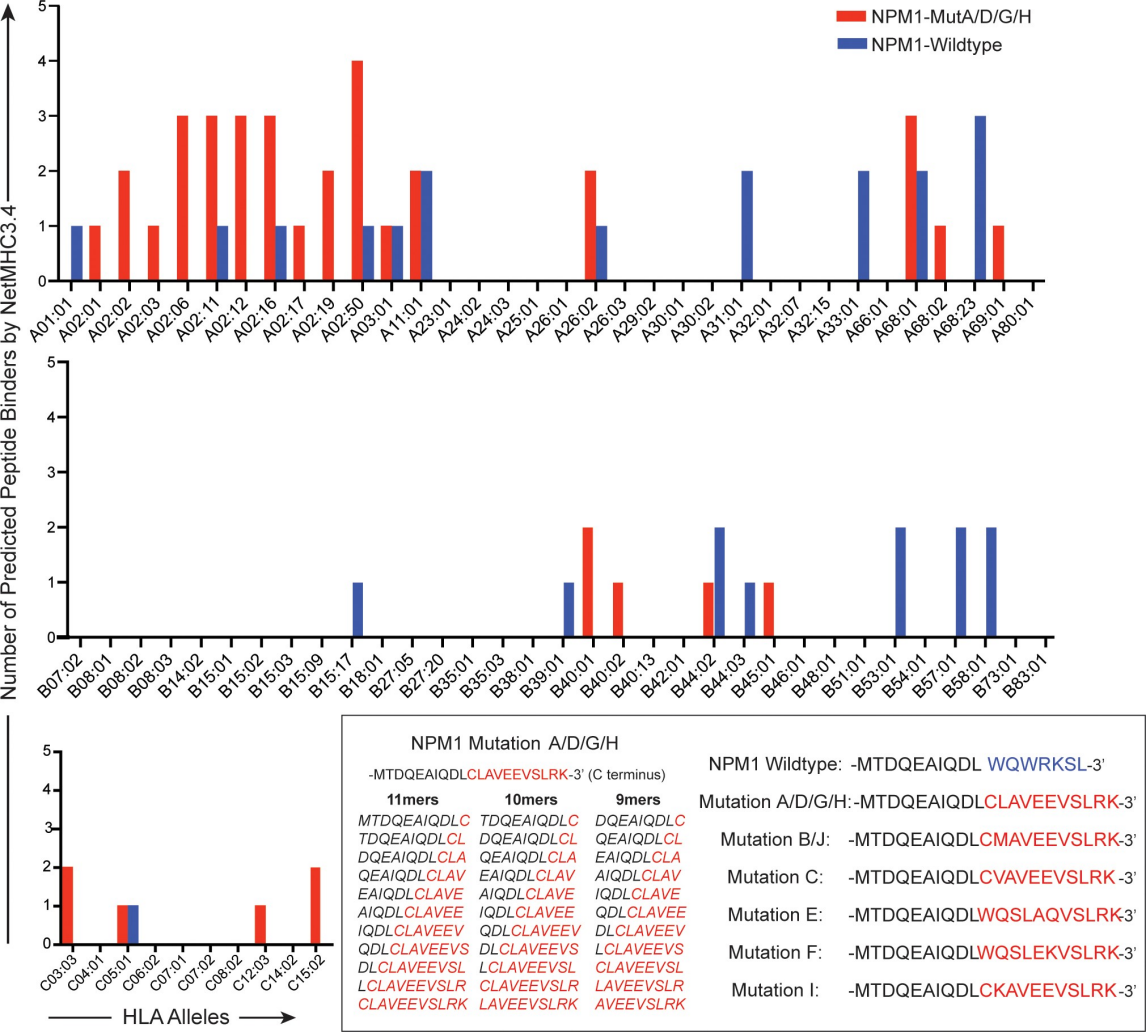
The number of peptides from common recurrent mutations that are predicted HLA Class I binders using NPM1 as example. From the potential 9-11mer peptides overlapping NPM1 mutation A/D/G/H, which contain a shared C terminal sequence, we evaluated the number of predicted HLA Class I binders, using available HLA-A, B, and C alleles in NetMHC3.4. Results were compared to the number of predicted HLA Class I binders from putative peptides from the corresponding wildtype NPM1 sequence. C-terminal peptide sequences from wildtype and mutant NPM1 are listed for reference (per nomenclature used by Falini et al[15] and Suzuki et al[28]).

We used NetMHC to predict HLA class I binding affinities for putative 9-11mer peptides spanning common recurrent AML mutations. We compared these predictions with those generated from the corresponding wildtype peptide sequences (Fig 1, S1 Fig). We review, as an example, findings for putative peptides from mutated NPM1. NPM1 mutations A, D, G, and H [15,28] (NPM1-MutA/D/G/H) are all predicted to result in a shared C-terminal amino acid residue sequence (MTDQEAIQDLCLAVEEVSLRK) that markedly differs from the wildtype sequence (MTDQEAIQDLWQWRKSL) (Fig 1). As has been previously described [29–31], the mutation bearing sequence AIQDLCLAV was predicted to strongly bind the HLA-A*02:01 allele (IC50, 97 nM), whereas no high affinity ligands were predicted to bind A*02:01 from the wildtype peptide sequence. The mutation-bearing sequence AVEEVSLRK, which is shared by most NPM1 mutations [15,28], was predicted to bind both HLA-A*03:01, a common allele across major ethnic groups in the US [19], and A*11:01. Several other peptide sequences from NPM1-MutA/D/G/H that were predicted to bind various HLA-A, B, and C alleles included MTDQEAIQDLC, IQDLCLAVEEV, DLCLAVEEVSL, QEAIQDLCLAV, EAIQDLCLAV, LCLAVEEVSL, CLAVEEVSLR, LAVEEVSLRK, QEAIQDLCL, DLCLAVEEV, CLAVEEVSL, and LAVEEVSLR. We further note that peptide sequences derived from the common recurrent mutations of DNMT3A, FLT3, KIT, RAS, and IDH2 yield predicted HLA-specific ligands, whereas IDH1 R132C/H did not produce as many predicted ligands (S1 Fig).

### AML HLA Class I and II Immunopeptidome analysis

**Fig 2:**
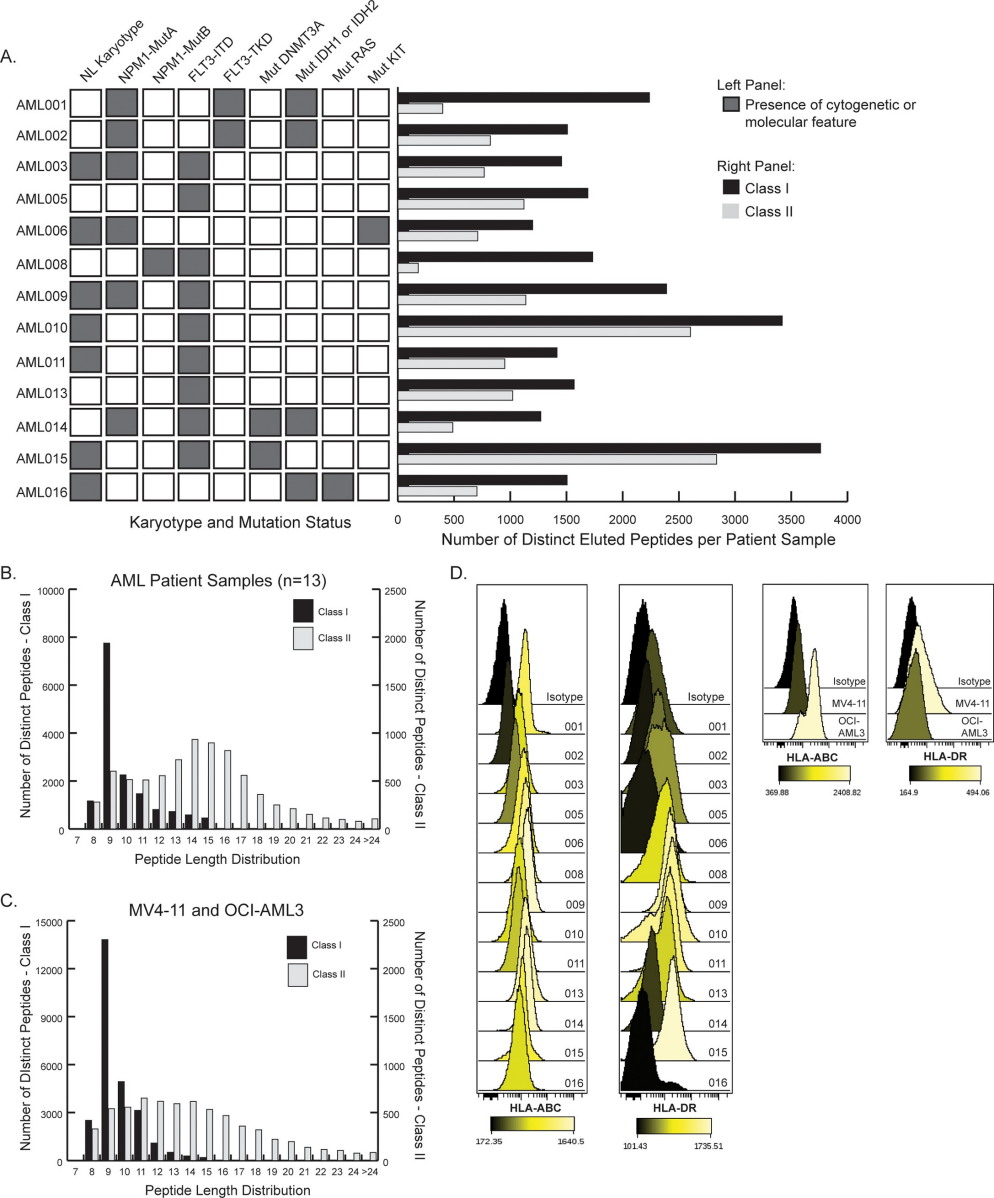
Number and length distribution of eluted peptides. (A) Cytogenetic and molecular features present in the thirteen patient samples are shaded dark gray on left side of panel. Right side of panel shows the number of distinct peptides eluted per sample from HLA Class I and Class II complexes. (B and C) Peptide length distribution from the combined peptide datasets of patient samples (B) and tumor cell lines (C). (D) HLA Class I and II expression by flow cytometry of patient samples (left) and cell lines (right).

We next empirically measured the HLA Class I and Class II (HLA-DR) immunopeptidomes of thirteen primary AML tumor samples (Table 1), with mass spectrometry using 1 X 10^8^ cells per MHC preparation. These data were used to evaluate whether endogenous HLA ligands spanning common recurrent AML mutations could be detected. Pan-HLA Class I and Class II HLA-DR immune complexes were captured in parallel experiments, rather than sequentially, to increase the sensitivity of our assay. Primary AML tumor samples were selected based on having known HLA haplotypes for HLA-A, B, C and HLA-DR, and at least one or more common recurrent mutations in either *NPM1*, *FLT3*, *DNMT3A*, *IDH1*, *IDH2*, *KIT*, or *RAS* from previous clinical evaluation. Known *NPM1* and *FLT3-ITD* mutations from patient samples were confirmed using sanger sequencing. More than half of the tumor samples had normal karyotypes; nearly half (6 of 13) bore *NPM1* mutation A and most (9 of 13) had *FLT3-ITD* mutations (Fig 2A), likely reflecting the increased frequency of banked tumor specimens from patients with high white blood cell counts. Nearly half the specimens were from patients with relapsed or refractory disease (6 of 13).

**Table 1:** Sample characteristics.

Mass Spec ID	Sample Disease Status	Peripheral WBC count 10^3^/μl (Blast %*)	Blast % from PBMC samples†	HLA Genotype Class I	HLA Genotype Class II
AML001	Relapsed	176 (97%)	93%	A*32 A*33 B*14 B*44 C*05 C*08	DRB1*01 DRB1*11
AML002	New Diagnosis	234 (95%)	82%	A*02 A*03 B*07 B*44 C*05 C*07	DRB1*04 DRB1*15
AML003	Refractory	52 (81%)	96%	A*03 A*03 B*07 B*07 C*07 C*07	DRB1*15 DRB1*15
AML005	Relapsed	228 (97%)	94%	A*03 A*24 B*07 B*35 C*04 C*07	DRB1*13 DRB1*14
AML006	New Diagnosis	207 (96%)	98%	A*25 A*31 B*18 B*48 C*08 C*12	DRB1*09 DRB1*15
AML008	Relapsed	37 (76%)	92%	A*01 A*26 B*14 B*55 C*03 C*08	DRB1*11 DRB1*11
AML009	New Diagnosis	162 (94%)	97%	A*01 A*02 B*27 B*57 C*01 C*06	DRB1*14 DRB1*15
AML0010	New Diagnosis	32 (35%)	67%	A*32 A*68 B*44 B*53 C*04 C*06	DRB1*11 DRB1*15
AML0011	New Diagnosis	153 (93%)	88%	A*24 A*34 B*35 B*53 C*04 C*06	DRB1*13 DRB1*14
AML0013	Relapsed	98 (94%)	60%	A*01 A*29 B*14 B*57 C*06 C*08	DRB1*07 DRB1*13
AML0014	Relapsed	62 (67%)	88%	A*24 A*31 B*51 B*58 C*03 C*14	DRB1*03 DRB1*10
AML0015	New Diagnosis	155 (66%)	84%	A*01 A*68 B*27 B*35 C*04 C*07	DRB1*01 DRB1*08
AML0016	New Diagnosis	18 (74%)	91%	A*01 A*24 B*55 B*57 C*03 C*06	DRB1*07 DRB1*13
OCI-AML3	NA	NA	NA	A*02 A*23 B*44 B*53 C*04 C*05	DRB1*04 DRB1*13
MV4-11	NA	NA	NA	A*03 A*68 B*14 B*18 C*08 C*15	DRB1*01 DRB1*13

*Percentage of peripheral blasts clinically reported.

†Percentage of blasts from PBMC specimens was determined using flow cytometry with dim/moderate CD45 versus low SSC-H for typical blast gate and high CD45 versus moderate SSC-H for myelomonocytic blast gate.

We identified a total of 20,169 distinct peptide sequences (12,406 peptides present only in the Class I dataset, 4,954 peptides present only in the Class II dataset, and 2,809 peptides present in both the Class I and II datasets) from all patient samples in this dataset (n = 13; estimated 1% FDR). Since we assayed patients’ PBMCs without further cell type enrichment, some of these peptides may have been presented by normal blood cells. However, the majority of specimens (11 of 13) had >80% blasts (Table 1), consistent with high tumor burdens. To try to further increase the sensitivity of mutant peptide detection, we also assessed the HLA immunopeptidome of two common AML cell lines using a higher cell count of 1 X 10^9^ cells per MHC preparation. These cell lines, OCI-AML3 and MV4-11, have been described to have *NPM1* mutation A [18] and *FLT3-ITD* [19] respectively. From the combined cell lines dataset, we identified a total of 31,734 distinct peptide sequences (25,212 peptides present only in the Class I dataset, 5,204 peptides present only in the Class II dataset, and 1,318 peptides present in both the Class I and II datasets).

The length distribution of Class I peptides measured from patient samples and cell lines followed the expected distribution, with a peak for 9mers and general range of 8-15mers (Fig 2B and 2C). Class II peptides distributed more broadly as expected (Fig 2B and 2C).

We evaluated Class I HLA-ABC and Class II HLA-DR expression by flow cytometry to compare HLA expression between primary tumor samples and to see if expression levels correlated with peptide recovery. The median fluorescent intensity (MFI) for HLA-ABC expression had less variability between patient samples (median MFI 1092 +/- std 416, range 290–1641), whereas HLA-DR expression had greater variability between samples (median MFI 587 +/- std 591, range 111–1736) (Fig 2D). While Class I expression did not correlate with the number of distinct peptides eluted from Class I from patients’ tumor samples (Pearson 0.05), there was a trend towards correlation between Class II HLA-DR expression and the number of distinct class II peptides eluted (Pearson 0.71) (S2 Fig). Comparing samples from newly diagnosed versus relapsed/refractory patients, we found that HLA expression was not significantly different between these groups but there was a trend towards a decreased number of distinct Class II peptides eluted from relapsed/refractory samples (S2 Fig).

**Fig 3:**
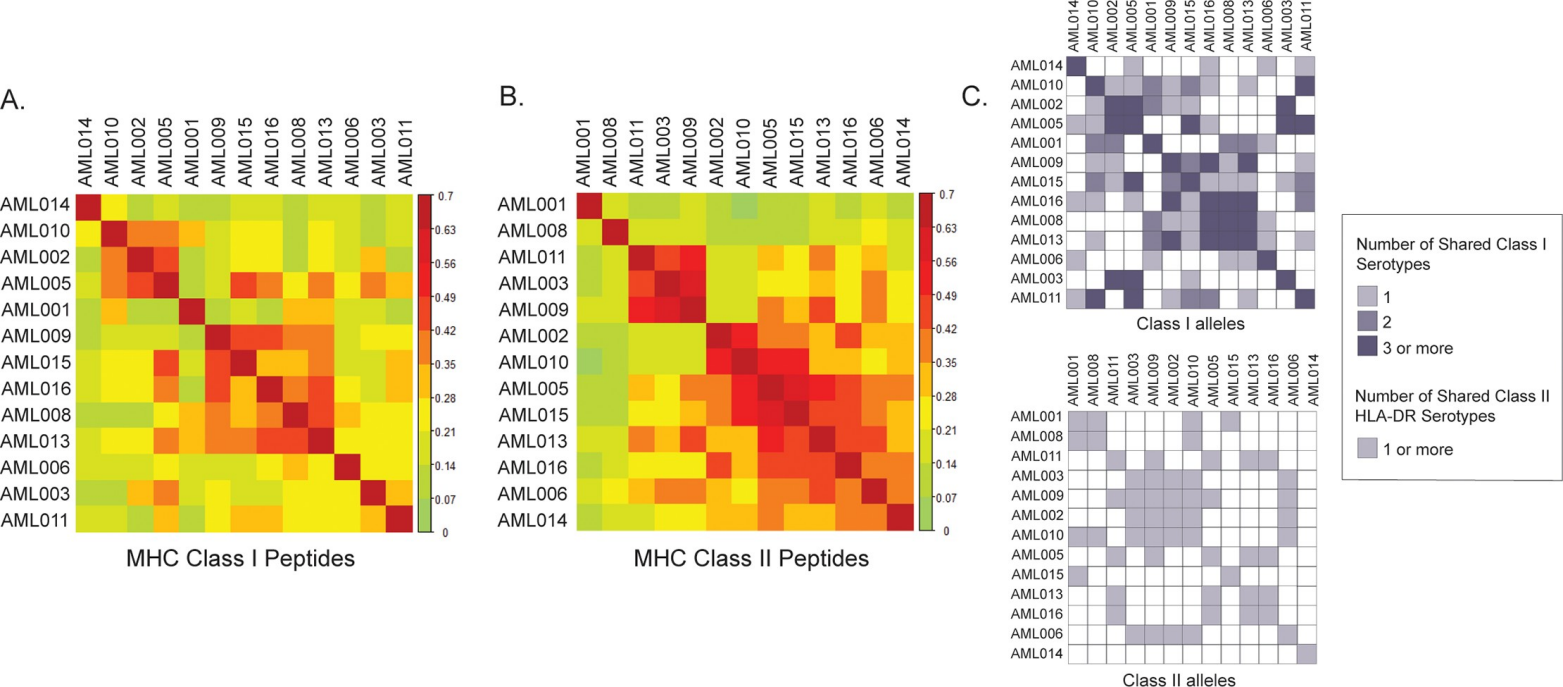
Comparison of peptide similarity between patient samples. Heatmaps based on Sorensen similarity coefficient comparing degree of similarity between peptides eluted from HLA Class I (A) and Class II (B) from patient samples. Clustering based on hierarchical cluster analysis. (C) Number of shared HLA Class I (above) and Class II DR (below) serotypes between patient samples.

We next evaluated the dataset of eluted, distinct peptides and their corresponding source genes/proteins in several ways including interpatient similarity and gene/protein ontology. Similar to our findings in mantle cell lymphoma [8], we observed considerable similarity between eluted peptides measured from patients with shared HLA serotypes and less similarity between patients with fewer HLA serotypes in common (Fig 3). The corresponding source proteins, however, were far more consistent between patients (S3 Fig) [8]. We also evaluated ontology of source genes from Class I and II peptides from patient tumor samples and cell lines using GOrilla [26,27] (S4 Fig). Similar to previous reports [32–34], we found that proteins presented by Class I reflected multiple cellular locations, including the nucleus, cytoplasm, and other membrane bound and non-membrane bound locations, whereas proteins presented by Class II appeared to have a more limited cellular location sampling that included vesicle, luminal associated and extracellular spaces (S4 Fig). These combined attributes gave us confidence in our dataset to next assess the presence of peptides from known leukemia associated antigens and recurrent AML mutations.

### Endogenous HLA ligands from source proteins of Leukemia associated antigens

**Fig 4:**
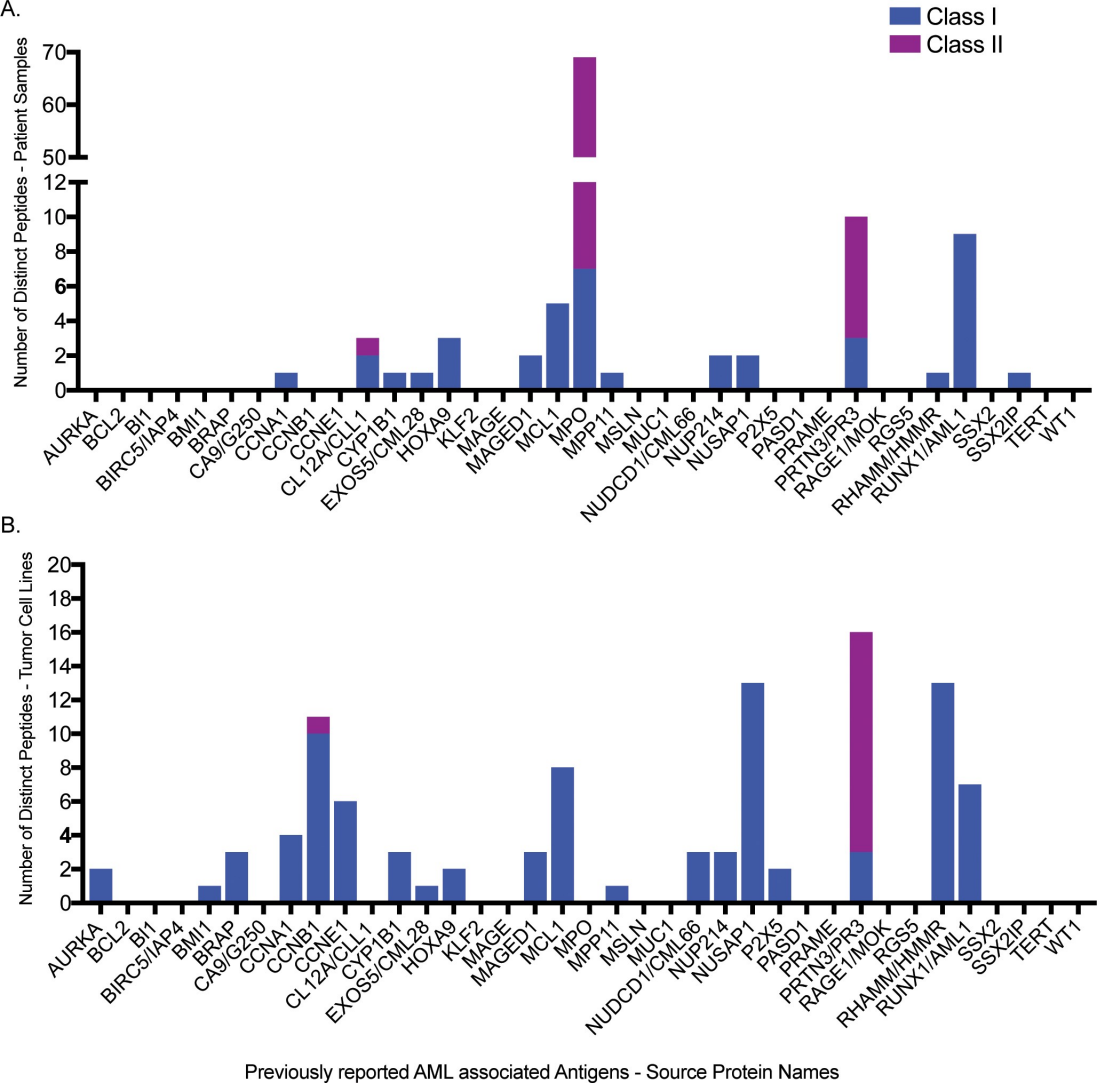
Number of peptides from source proteins of leukemia associated antigens. The number of distinct Class I and Class II peptides from (A) patient samples and (B) cell lines per source proteins of previously reported leukemia associated antigens.

Several LAAs have been described in the literature [2,3] such as WT1 [35,36] and CCNA1 [37]. We evaluated the Class I and II immunopeptidomes from patient samples and cell lines for source proteins of previously reported LAAs [2,3,14] (Fig 4, S2 Table). While we found several peptides from LAA source proteins such as PRTN3/PR3 and MPO, we did not find any from others such as WT1 or BIRC5 in this dataset. Several of the peptides from LAAs have not been previously reported to be eluted from primary AML samples to our knowledge, such as SLSEIVPC(cys)L, a Class I peptide from CCNA1 found in AML009 (S2 Table).

### Identification of endogenous mutated HLA ligands

**Fig 5:**
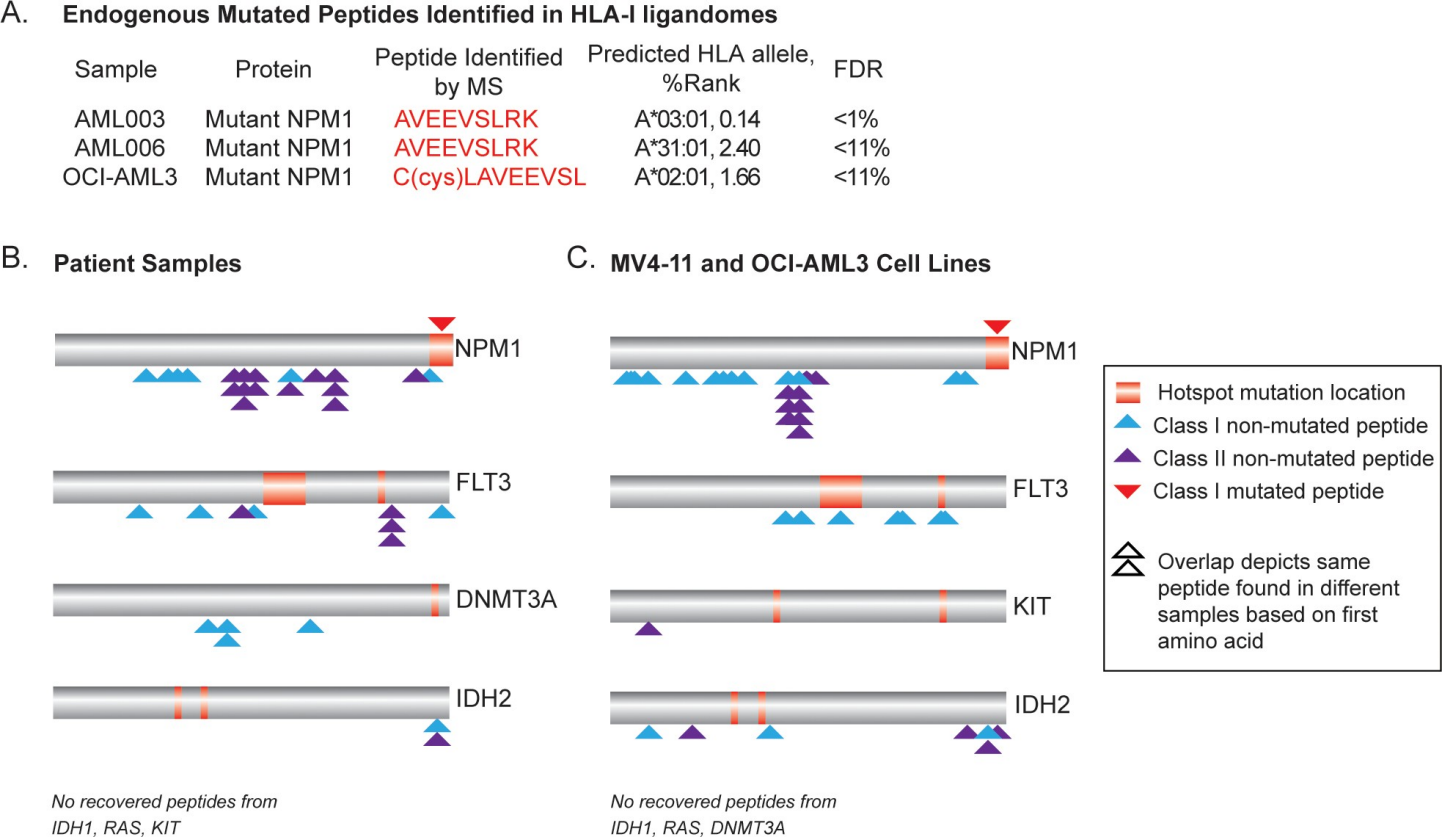
Endogenous mutated peptides from NPM1 identified by MS. (A) List of endogenous mutated Class I peptides that were identified by MS. The most likely predicted HLA binder was selected based on %rank by NetMHCpan4.0 based on the HLA haplotype for each sample. (B and C) Depiction of the protein location of eluted mutation bearing and non-mutation bearing peptides, in relation to recurrent AML hotspot mutations in proteins of interest, from patient samples (B) and cell lines (C).

We next searched the MS data against a database combining the human proteome with mutation sequences from common recurrent mutations (from NPM1, FLT3-TKD, RAS, KIT, DNMT3A, IDH1/2) and the unique FLT3-ITD sequences identified in patient samples. Using a stringent 1% FDR threshold, we identified an endogenous 9mer peptide from mutated NPM1, AVEEVSLRK, from one patient sample (AML003) in Class I immunopeptidome analysis (Fig 5A). Following a strategy described by Bassani-Sternberg et al.[7], we considered identifications meeting a less stringent FDR threshold to increase the sensitivity with which we could measure mutant peptides. With a threshold of less than 11% applied to Class I ligand data, we identified the same AVEEVSLRK peptide from another patient sample (AML006) and also a cysteinylated 9mer peptide from NPM1, C(cys)LAVEEVSL from OCI-AML3 (Fig 5 and S3 Table). Both peptides’ identities were confirmed using synthetic peptides (S5 Fig). Based on the HLA haplotypes of the samples in which the peptides were identified, we determined that AVEEVSLRK is likely presented by A*03:01 in AML003 and A*31:01 in AML006, with C(cys)LAVEEVSL likely being presented by A*02:01 in OCI-AML3 (Fig 5). We also identified several short length NPM1 mutation-bearing peptides from Class II immunopeptidome analysis, including AVEEVSLRK, LAVEEVSLRK, VEEVSLRK, and AVEEVSLR (S3 Table). Although short length ligands have been observed in Class II immunopeptidome studies [7,33] their significance remains poorly understood.

In addition to identifying mutation-bearing ligands, we also evaluated for the presence of non-mutation bearing ligands from proteins that can be recurrently mutated in AML (Fig 5 and Table 2). Among the proteins of interest (NPM1, FLT3, DNMT3A, IDH1, IDH2, KIT, and RAS), non-mutation bearing ligands from NPM1 were the most frequent in both patient tumor samples and cell lines, including ligands close to or corresponding to where hotspot mutations occur (EAIQDLWQW and MTDQEAIQDLWQWR). We did not measure any ligands from the proteins IDH1 or RAS in this dataset. Whether the processing and presentation of non-mutation bearing HLA ligands from wildtype regions increases the likelihood of mutation-bearing peptides being processed and presented from the same region remains to be further explored. While the cytoplasmic localization of NPM1 mutant proteins may potentially impact processing and presentation of both mutation bearing and non-bearing peptides from the mutant protein, in this dataset most of the NPM1 peptides were eluted from NPM1 wildtype samples (Table 2).

**Table 2:** Non-mutated peptides eluted from HLA Class I and II from patient samples and cell lines from source proteins of interest.

Source Protein	HLA	Sample	Mutation Status of Sample	Eluted Peptide
NPM1				
	Class I			
		AML010	NPM1 Wildtype	DDEEAEEKAPVKK
		AML015	NPM1 Wildtype	GGFEITPPVVLR
		MV411	NPM1 Wildtype	GGFEITPPVVLR
		AML015	NPM1 Wildtype	GFEITPPVVLR
		MV411	NPM1 Wildtype	FEITPPVVLR
		AML015	NPM1 Wildtype	EITPPVVLR
		MV411	NPM1 Wildtype	EITPPVVLR
		MV411	NPM1 Wildtype	ITPPVVLR
		AML003	NPM1 Mutated	SPIKVTLATL
		OCIAML3	NPM1 Mutated	C[119.00]ELKADKDY
		OCIAML3	NPM1 Mutated	C[119.00]ELKADKDYHF
		OCIAML3	NPM1 Mutated	KADKDYHF
		OCIAML3	NPM1 Mutated	KFINYVKNCF
		MV411	NPM1 Wildtype	VEAKFINY
		MV411	NPM1 Wildtype	DENEHQLSL
		MV411	NPM1 Wildtype	SGKRSAPGGGSKVPQ
		MV411	NPM1 Wildtype	RSAPGGGSKVPQK
		MV411	NPM1 Wildtype	VEAEAMNY
		AML010	NPM1 Wildtype	EAIQDLWQW*
	Class II			
		AML010	NPM1 Wildtype	DDEEAEEKAPVKK
		AML005	NPM1 Wildtype	LSISGKRSAPGGGSKVPQ
		AML006	NPM1 Mutated	LSISGKRSAPGGGSKVPQ
		AML006	NPM1 Mutated	SISGKRSAPGGGSKVPQ
		AML013	NPM1 Wildtype	SISGKRSAPGGGSKVPQ
		AML015	NPM1 Wildtype	SISGKRSAPGGGSKVPQ
		AML005	NPM1 Wildtype	SGKRSAPGGGSKVPQKKVKL
		AML010	NPM1 Wildtype	SGKRSAPGGGSKVPQKKV
		MV411	NPM1 Wildtype	SGKRSAPGGGSKVPQKKV
		MV411	NPM1 Wildtype	SGKRSAPGGGSKVPQ
		OCIAML3	NPM1 Mutated	SGKRSAPGGGSKVPQ
		MV411	NPM1 Wildtype	RSAPGGGSKVPQKKV
		MV411	NPM1 Wildtype	RSAPGGGSKVPQK
		MV411	NPM1 Wildtype	RSAPGGGSKVPQ
		MV411	NPM1 Wildtype	RSAPGGGSKVP
		MV411	NPM1 Wildtype	SAPGGGSKVPQ
		AML010	NPM1 Wildtype	SIRDTPAKNAQK
		MV411	NPM1 Wildtype	KKVKLAADEDDDDD
		AML010	NPM1 Wildtype	SNQNGKDSKPSSTPRSKGQESF
		AML010	NPM1 Wildtype	SNQNGKDSKPSSTPRSKGQESFK
		AML010	NPM1 Wildtype	SNQNGKDSKPSSTPRSKGQESFKK
		AML005	NPM1 Wildtype	MTDQEAIQDLWQWR*
FLT3				
	Class I			
		AML011	FLT3 Mutated	EAIKGFLVK
		AML001	FLT3 Mutated	HELFGTDI
		AML001	FLT3 Mutated	KAYPQIRC[119.00]TW
		AML001	FLT3 Mutated	RPFSREMDL
		OCIAML3	FLT3 Wildtype	AEASASQASC[119.00]F
		MV411	FLT3 Mutated	DIMSDSNYVVR*
		OCIAML3	FLT3 Wildtype	IMSDSNYVV*
		MV411	FLT3 Mutated	EITEGVWNR
		MV411	FLT3 Mutated	FRYESQLQM*
		MV411	FLT3 Mutated	SSMPGSREV
		OCIAML3	FLT3 Wildtype	TEIFKEHNF
	Class II			
		AML005	FLT3 Mutated	DSNYVVRGNARLPVK*
		AML009	FLT3 Mutated	DSNYVVRGNARLPVK*
		AML011	FLT3 Mutated	DSNYVVRGNARLPVK*
		AML001	FLT3 Mutated	ITEGVWNRKANRKVFG
DNMT3A				
	Class I			
		AML003	DNMT3A Wildtype	ATYNKQPMY
		AML001	DNMT3A Wildtype	EVLQVASSR
		AML011	DNMT3A Wildtype	EVLQVASSR
		AML006	DNMT3A Wildtype	GTYGLLRRR
IDH2				
	Class I			
		AML002	IDH2 Mutated	LDTIKSNLDRALGRQ
		MV411	IDH2 Wildtype	ADKRIKVAKPV
		MV411	IDH2 Wildtype	HGDQYKATDFV
		MV411	IDH2 Wildtype	KLNEHFLNT
	Class II			
		AML002	IDH2 Mutated	LDTIKSNLDRALGRQ
		MV411	IDH2 Wildtype	LDTIKSNLDRALGRQ
		MV411	IDH2 Wildtype	GLPNRDQTDDQVTIDS
		MV411	IDH2 Wildtype	KLNEHFLNT
		MV411	IDH2 Wildtype	VESGAMTKDL
KIT				
	Class II			
		MV411	KIT Wildtype	ENKQNEWITEKAEATNTG

Non-mutation bearing HLA Class I and II peptides eluted from proteins of interest that are recurrently mutated in AML using FDR 1% are listed.

*Overlaps or near to hotspot mutation location of protein.

## Discussion

Endogenous mutation-bearing HLA ligands from primary human tumor samples have been successfully identified in melanoma [7,9] and lymphoma [8]. In this study, we searched for the HLA presentation of mutation-bearing peptides from recurrent mutations commonly shared between patients with AML, as such ligands would be specific to tumors and personal, yet also provide shared anti-tumor targets for potential future immunotherapy. We identified over 47,000 distinct HLA ligands and report the identification of endogenous mutation-bearing Class I peptides from mutated NPM1 (AVEEVSLRK in two patient samples and C(cys)LAVEEVSL in OCI-AML3). To our knowledge, there have only been two other studies of AML membrane derived HLA immunopeptidome analysis. The first study evaluated the HLA Class I and II immunopeptidome of primary AML tumor samples with a focus on leukemia-associated ligands [14]. A recently published second study evaluated the HLA Class I immunopeptidome of twelve primary AML samples for mutated NPM1 ligands [38]. Similar to our study, they reported finding the Class I presentation of AVEEVSLRK and CLAVEEVSL; additionally they found VEEVSLRK, AVEEVSLR, CLAVEEVSLRK [38].

Our findings have the potential for therapeutic translation. *NPM1* is mutated in approximately one-third of patients with adult AML [11]. Approximately 30–70% of patients with *NPM1* mutated AML have disease relapse within five years [39–41], depending on factors such as age and the presence of concurrent *FLT3-ITD* mutations. The majority of *NPM1* mutations are due to mutations A, B and D, with mutation A accounting for around 70–80% of all *NPM1* mutations [42,43]. The peptide sequence CLAVEEVSL is shared between mutations A, D, G, and H [28,42], while the sequence AVEEVSLRK is shared between the vast majority of NPM1 mutations, including A, B, C, D, G, and H [42]. CLAVEEVSL and AVEEVSLRK are predicted to bind and have the correct anchor residues for A*02:01 and A*03:01 respectively. Peptide AVEEVSLRK is also a strong predicted binder to A*11:01 and a weak predicted binder to A*30:01, A*66:01 and A*68:01 by NetMHCpan4.0. Using the Allele Frequency Net Database, A*03:01 has been reported to occur in around 24% and 21% in a population of African Americans and Caucasian Americans respectively [44]. A*02:01 has been reported to occur in around 40–50% of Caucasian Americans [44]. Kuzelova et al., compared HLA Class I frequencies in patients with AML compared to normal individuals [31]. Interestingly, they found that several HLA allele groups were less frequently found in NPM1 mutated patients (including statistical significance for B*07, B*18, and B*40 and a trend for A*03, A*11, B*39, C*03, and C*07) [31]. Additionally, they found that amongst patients with mutated NPM1, those with at least one of these types of alleles had overall survival advantage. This work suggests that the HLA haplotype presented by a tumor in addition to the somatic mutations a tumor has, may influence disease outcomes, potentially through immune interactions. Several studies have supported the general immunogenicity of NPM1 from both mutated and nonmutated peptides [29–31,45,46]. Greiner et al., found that the synthetic peptides AIQDLCLAV and AIQDLCVAV, which are predicted A2+ binders, elicit *in vitro* CD8+ T cell responses in both healthy donors and AML patients [30]. Their group also found a statistically significant increase in PD-L1 expression in the leukemic stem cell fraction of *NPM1* mutated AML compared to wildtype [47].

There are several potential therapeutic strategies to target the endogenous HLA presentation of mutated NPM1. Mutated peptides can be utilized to identify neoantigen specific, HLA restricted T cells and TCR sequences [38]. TCR sequences optimally recognizing the mutated NPM1-HLA complex may be used to transduce T cells from patients to derive AML specific cell therapy for patients with this shared mutation. Using a similar method, two recent studies are evaluating peripheral blood lymphocytes transduced with murine TCR recognizing the recurrent Ras mutation G12V in HLA-A*11:01 patients with solid tumors (NCT03190941, NCT03745326). T cells from patients or from HLA matched donors in the allogeneic transplant setting may also be stimulated *ex vivo* with NPM1 mutated peptides to enrich for neoantigen specific T cells followed by adoptive T cell therapy. A recent study is currently evaluating a similar strategy by stimulating donor-derived T cells with tumor associated antigens for AML and MDS followed by infusion at least 30 days after allogeneic stem cell transplant (NCT02494167). Another strategy targeting mutant NPM1 in AML would be to utilize NPM1 mutated peptides as part of a vaccination approach in combination with checkpoint inhibitors to stimulate an endogenous anti-tumor response.

In our next steps, we plan to evaluate patient and healthy donor samples to identify mutant NPM1 specific T cells followed by functional analysis for anti-tumor cytolytic ability and specificity which may help derive future cell therapy approaches. Additionally, the clinical relevance of neoantigen-recognizing allogeneic T cells in the context of hematopoietic cell transplant (HCT) remains poorly characterized. As HCT is potentially curative in AML, characterizing the presence and function of endogenous donor derived neoantigen-recognizing T cells may lead to novel therapeutic strategies. It will also be important to characterize whether HLA haplotype in the context of neoantigen presentation impacts outcomes in the allogeneic transplant setting. In summary, our identification of endogenous HLA ligands from mutated NPM1, which is one of the most frequently mutated proteins in AML, supports exploration of immunotherapy against this shared target.

## Supporting information

S1 Fig Predicted HLA Binders from common recurrent AML mutations. The number of predicted HLA binders from the potential 9-11mer peptides overlapping common recurrent mutations of AML and their corresponding wildtype regions were plotted using available HLA-A, B, and C alleles in NetMHC3.4. The number of predicted HLA Class I binders are shown for DNMT3A (A), FLT3-D835 (B), IDH1 (C), IDH2 (D), Ras (E, F, G), and KIT (H).
(TIF)

S2 Fig HLA expression by flow cytometry and comparison to peptide elution. (A) Gating strategy depicted using representative sample from AML009. (B) HLA median fluorescent intensity (MFI) versus number of distinct eluted peptides per each patient sample for Class I (left) and Class II DR (right). (C-D) Comparison of HLA Class I or II MFI in newly diagnosed versus relapsed/refractory samples (C) and in NPM1 mutated versus unmutated samples (D). (E-F) Comparison of the number of distinct eluted peptides per patient sample from HLA Class I or Class II in newly diagnosed versus relapsed/refractory samples (E) and in NPM1 mutated versus unmutated samples (F) (C-F, median with 95% confidence intervals shown, analysis done using Mann Whitney two tailed testing).
(TIF)

S3 Fig Similarity of source proteins of eluted peptides between patient samples. Heatmaps based on Sorensen similarity coefficient comparing degree of similarity between source proteins representing the eluted peptides from HLA Class I (A) and Class II (B), from patient samples. Clustering based on hierarchical cluster analysis.
(TIF)

S4 Fig Source genes from eluted peptides were analyzed for gene ontology by cellular component using GOrilla. Cellular component analyses are depicted for patient samples (A, Class I; B, Class II) and cell lines (C, Class I; D, Class II).
(DOCX)

S5 Fig Comparison of spectra between synthetic and endogenous mutated HLA Class I peptides from mutated NPM1. Spectra shown for (A) AVEEVSLRK and (B and C) C(cys)LAVEEVSL.
(TIF)

S1 File Supplemental materials and methods. (DOCX)

S2 File List of peptide predicted binding affinity from NetMHC3.4 for peptides of interest. (XLSX)

S3 File Calculations for peptide counts and flow cytometry data. (XLSX)

S1 Table Common recurrent AML mutations of interest. Common recurrent AML mutations of interest with their frequency reported in literature and origination of clinical mutation data annotated for patient samples in study.
(DOCX)

S2 Table List of eluted peptides per source proteins of previously published leukemia associated antigens. (A) List of eluted HLA Class I and Class II peptides from patient samples (n = 13) per source proteins of previously published leukemia associated antigens. The number of distinct Class I or II peptides in the combined data set derived from patient samples were counted. (B) List of eluted HLA Class I and Class II peptides from tumor cell lines (n = 2) per source proteins of previously published leukemia associated antigens. The number of distinct Class I or II peptides in the combined data set derived from the two cell lines were counted.
(DOCX)

S3 Table Peptides from recurrent mutations. Comparison of use of FDR 1% versus <11% for analysis of Class I (A) and Class II (B) eluted peptides for to identify peptides from recurrent mutations from patient samples and cell lines.
(DOCX)
