# Supplementary figures and images for: Acute myeloid leukemia immunopeptidome reveals HLA presentation of mutated nucleophosmin
Source: PLoS One. 2019 Jul 10;14(7):e0219547. doi: 10.1371/journal.pone.0219547 (PMC6619824; doi:10.1371/journal.pone.0219547)

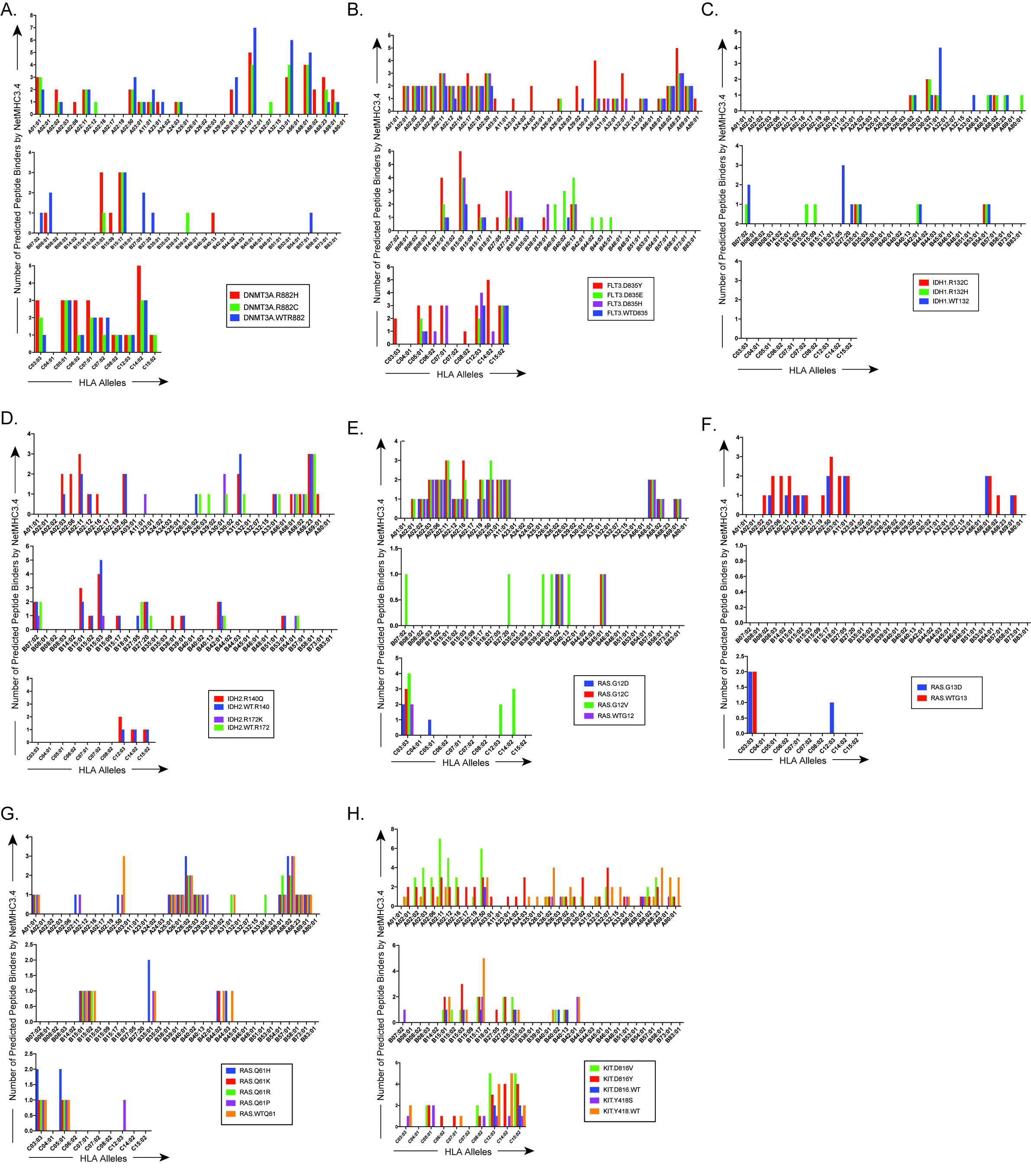

Supplement: S1 Fig — The number of predicted HLA binders from the potential 9-11mer peptides overlapping common recurrent mutations of AML and their corresponding wildtype regions were plotted using available HLA-A, B, and C alleles in NetMHC3.4. The number of predicted HLA Class I binders are shown for DNMT3A (A), FLT3-D835 (B), IDH1 (C), IDH2 (D), Ras (E, F, G), and KIT (H). (TIF) [file pone.0219547.s001.tif]

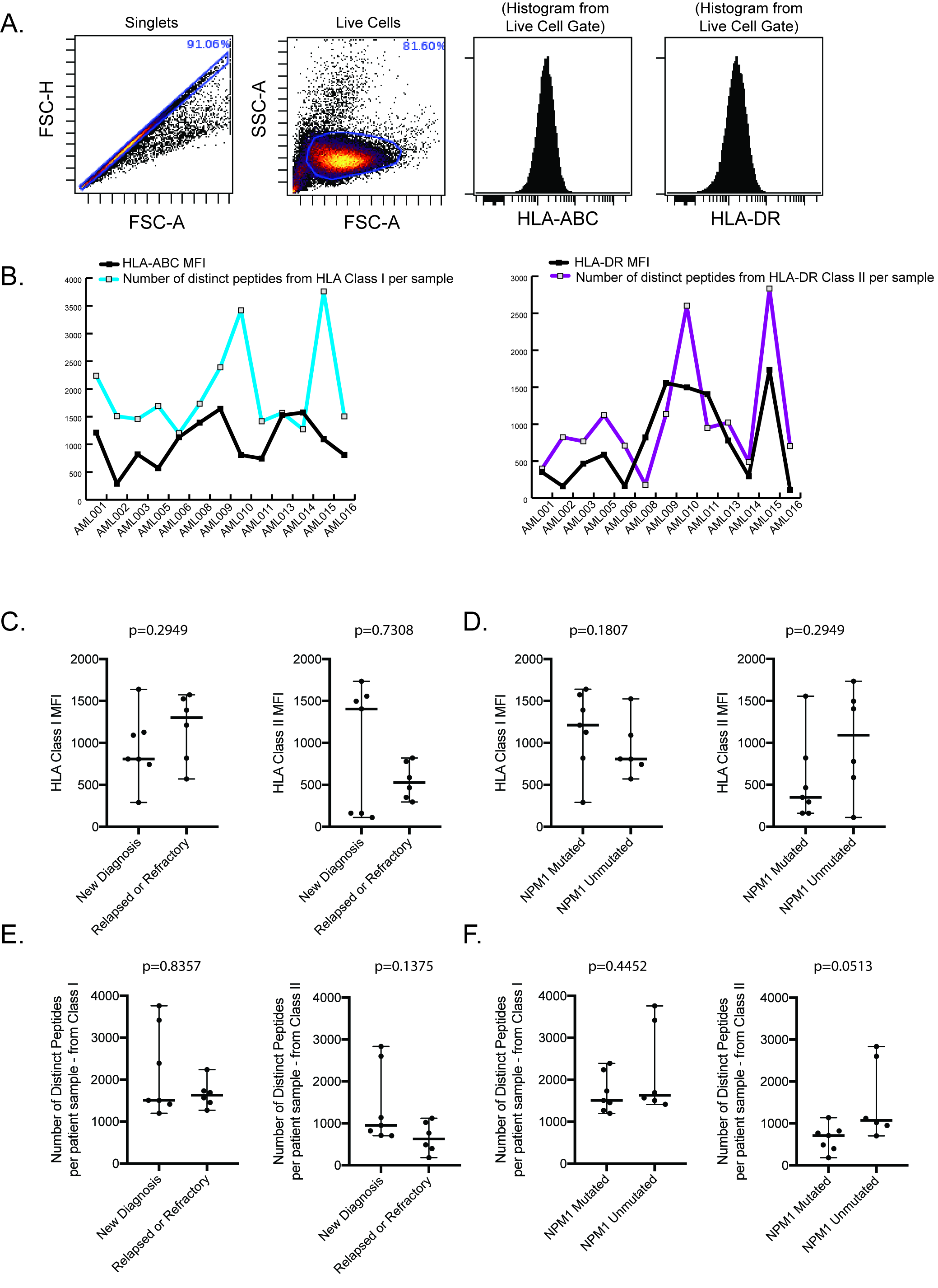

Supplement: S2 Fig — (A) Gating strategy depicted using representative sample from AML009. (B) HLA median fluorescent intensity (MFI) versus number of distinct eluted peptides per each patient sample for Class I (left) and Class II DR (right). (C-D) Comparison of HLA Class I or II MFI in newly diagnosed versus relapsed/refractory samples (C) and in NPM1 mutated versus unmutated samples (D). (E-F) Comparison of the number of distinct eluted peptides per patient sample from HLA Class I or Class II in newly diagnosed versus relapsed/refractory samples (E) and in NPM1 mutated versus unmutated samples (F) (C-F, median with 95% confidence intervals shown, analysis done using Mann Whitney two tailed testing). (TIF) [file pone.0219547.s002.tif]

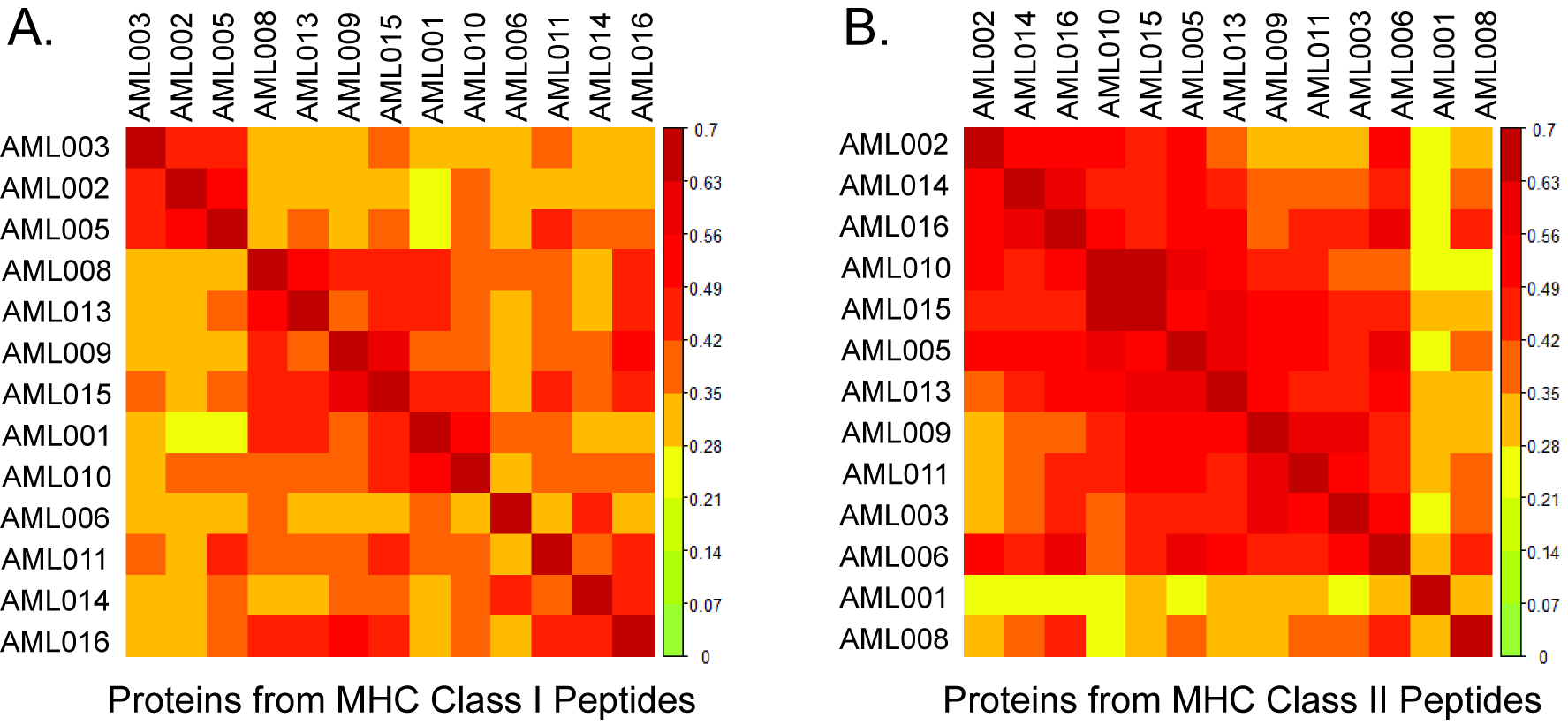

Supplement: S3 Fig — Heatmaps based on Sorensen similarity coefficient comparing degree of similarity between source proteins representing the eluted peptides from HLA Class I (A) and Class II (B), from patient samples. Clustering based on hierarchical cluster analysis. (TIF) [file pone.0219547.s003.tif]

Figure S4A.


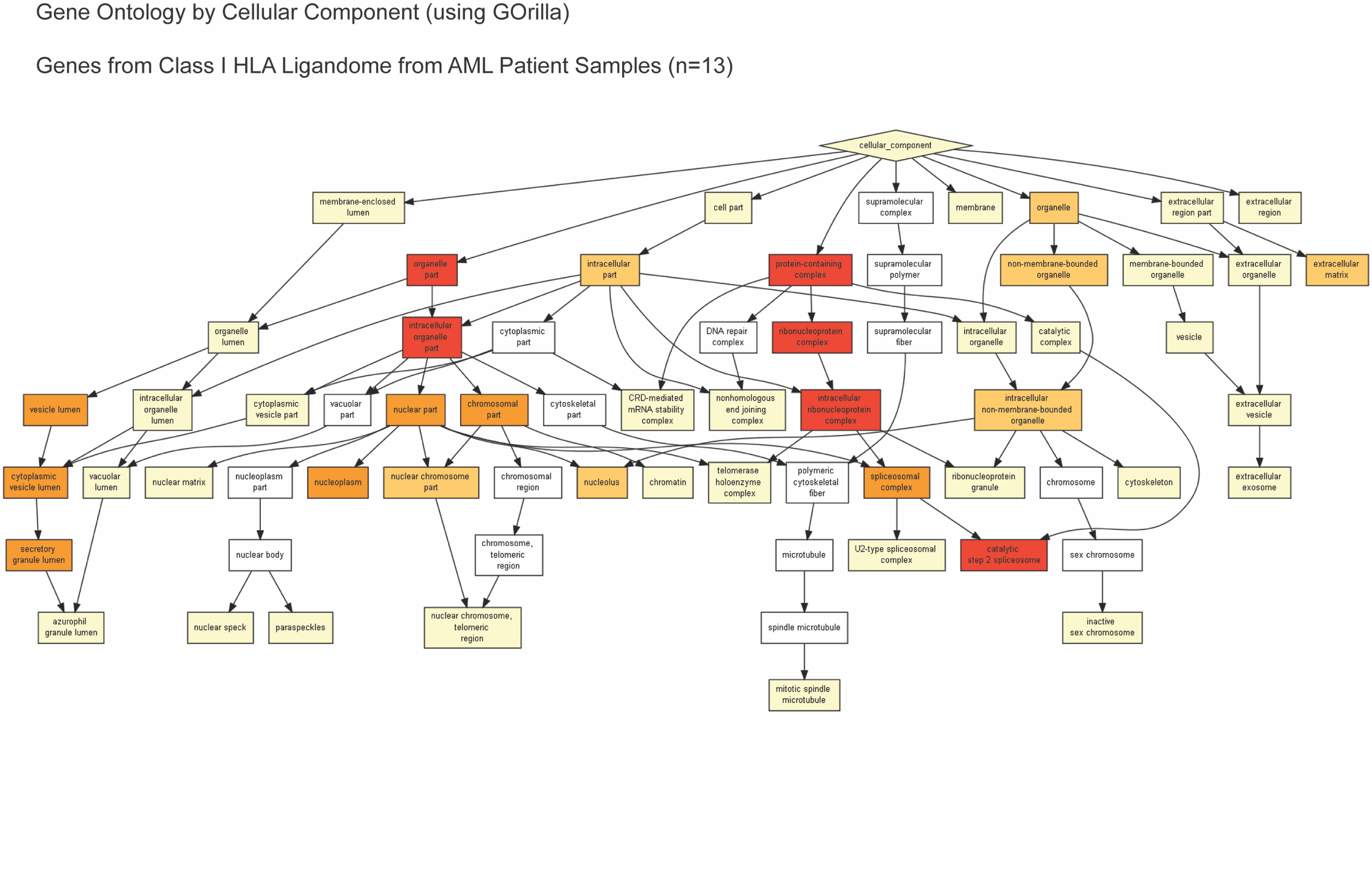


Figure S4B.


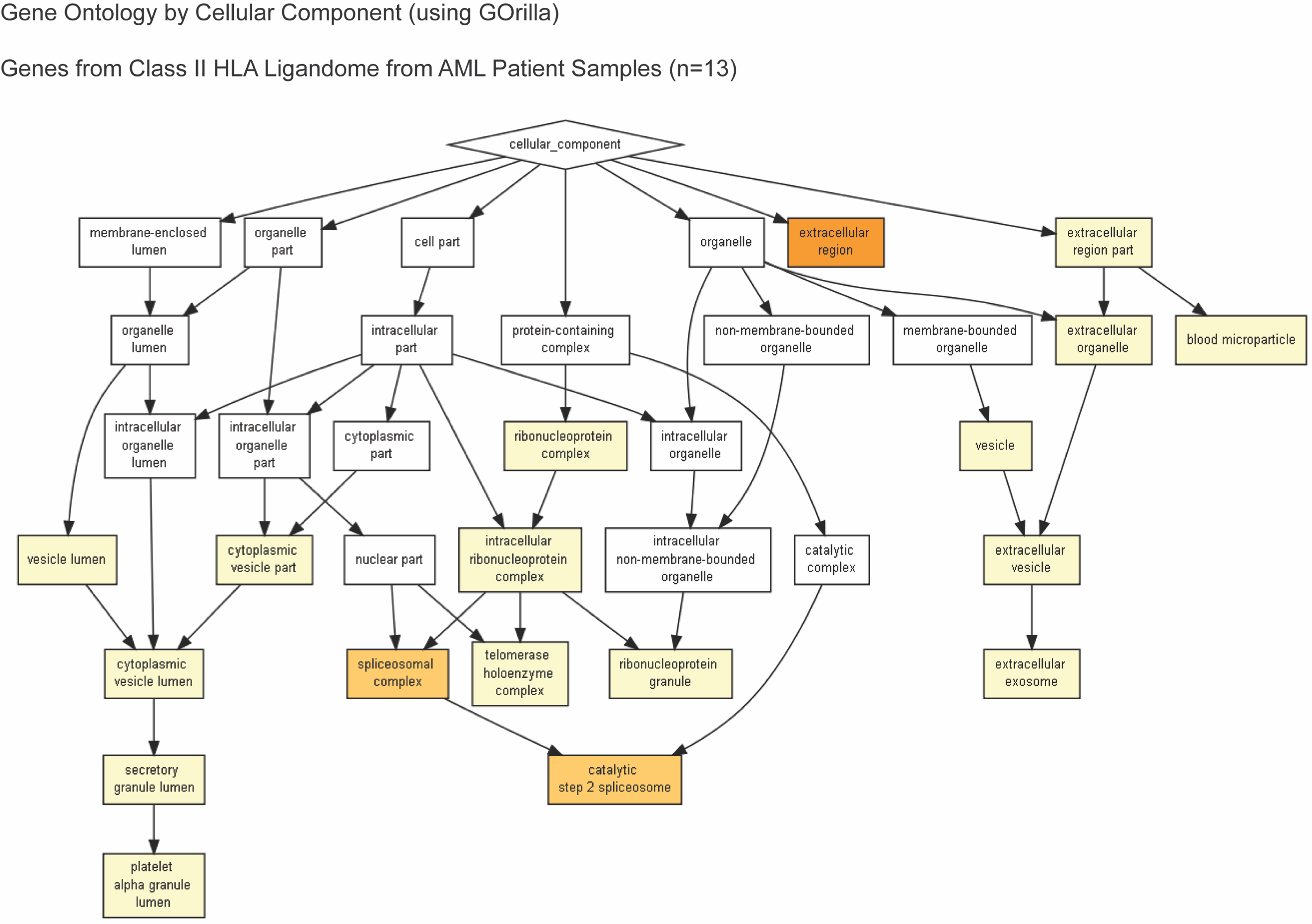


Figure S4C.


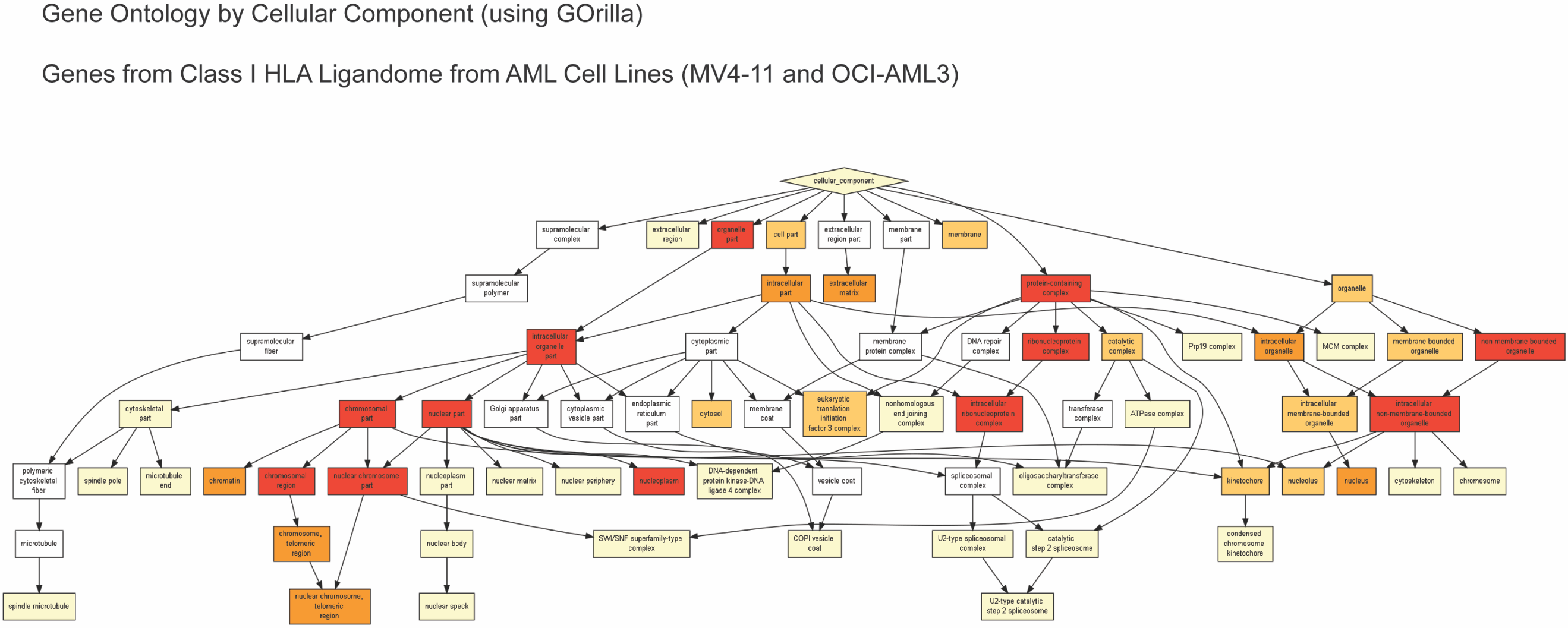


Figure S4D.


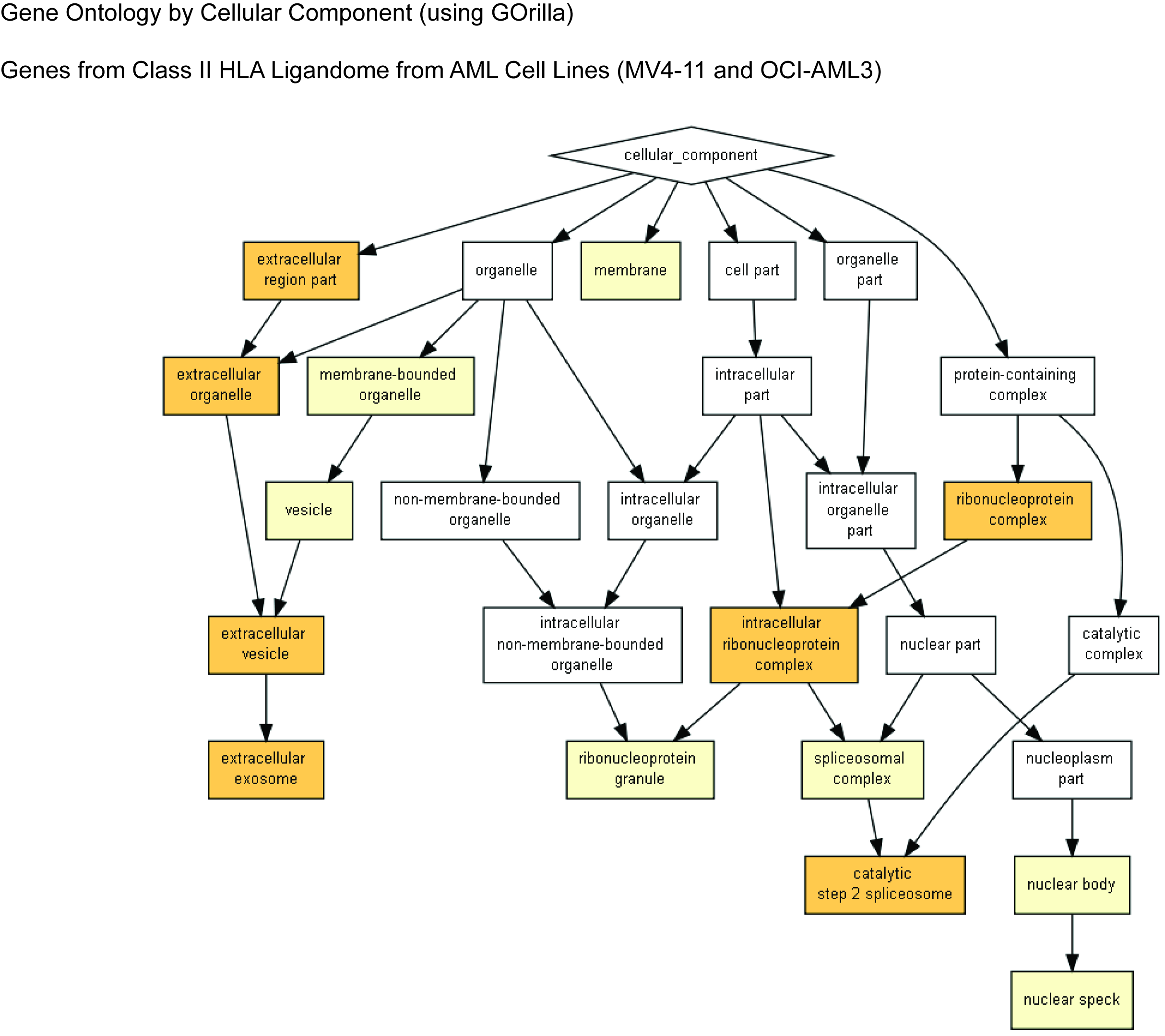

Supplement: S4 Fig — Cellular component analyses are depicted for patient samples (A, Class I; B, Class II) and cell lines (C, Class I; D, Class II). (DOCX) [file pone.0219547.s004.docx]

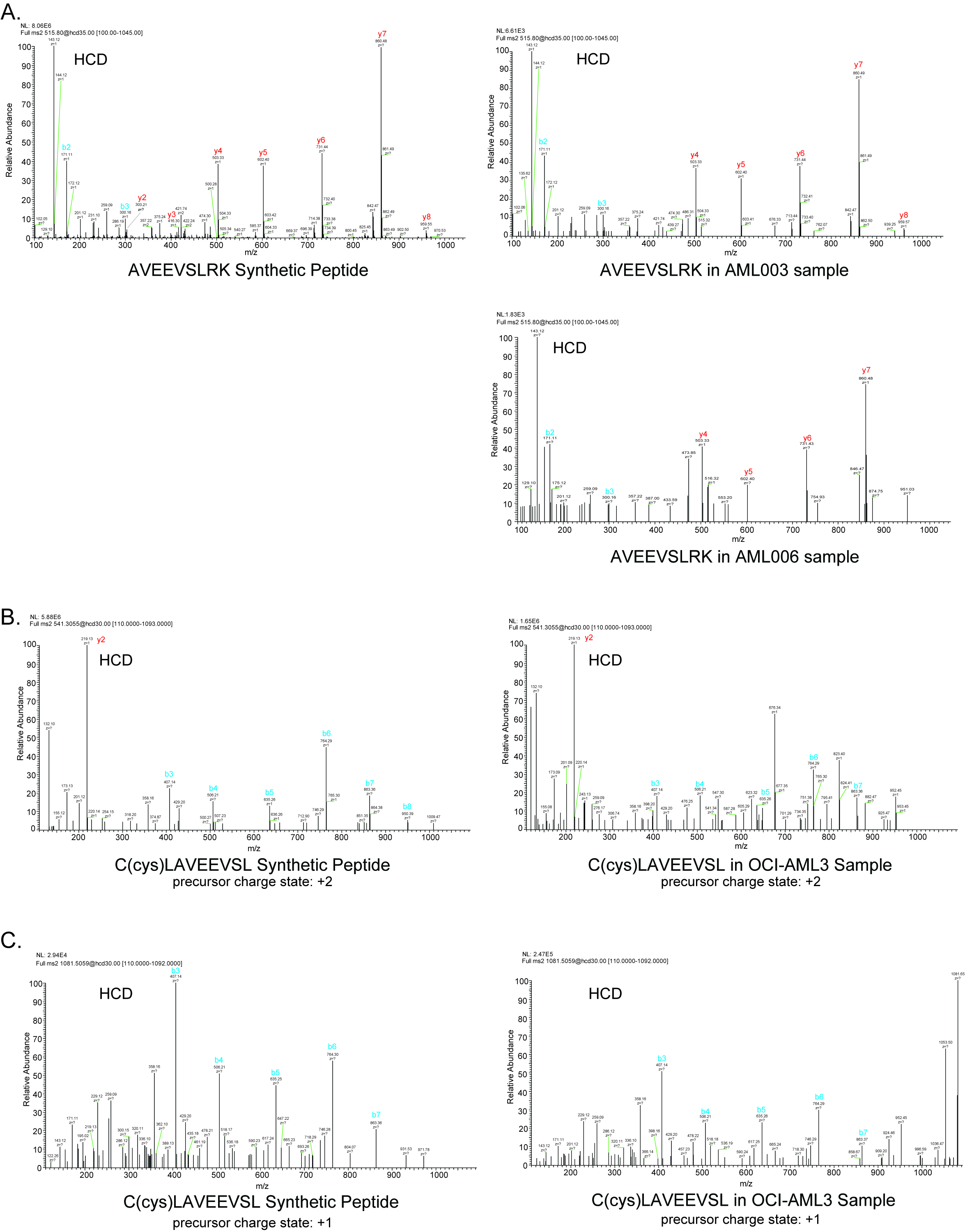

Supplement: S5 Fig — Spectra shown for (A) AVEEVSLRK and (B and C) C(cys)LAVEEVSL. (TIF) [file pone.0219547.s005.tif]
