# Supplementary material for: Acute myeloid leukemia immunopeptidome reveals HLA presentation of mutated nucleophosmin
Source: PLoS One. 2019 Jul 10;14(7):e0219547. doi: 10.1371/journal.pone.0219547 (PMC6619824; doi:10.1371/journal.pone.0219547)
Supplement: S1 File — (DOCX) [file pone.0219547.s006.docx]

**S1 File. Supplemental Materials and Methods**

**Mutation Analysis**

Genomic DNA was extracted from patient samples using the Qiagen Allprep DNA kit and stored in -80°C till use. PCR primers were obtained from the Stanford PAN facility or Elim Biopharm (Hayward, CA) and used at a working solution of 10μM. For FLT3-ITD analysis, the following primers were used to cover exon 14 and 15: FLT3 forward primer: 5’-GCAATTTAGGTATGAAAGCCAGC-3’ and FLT3 reverse primer: 5’-CTTTCAGCATTTTGACGGCAACC-3’. For NPM1 mutation analysis, the following primers were used: NPM1 forward primer: 5’-TTAACTCTCTGGTGGTAGAATGAA-3’ and NPM1 reverse primer: 5’-CAAGACTATTTGCCATTCCTAAC-3’. Initial cloning from genomic DNA generally used the following PCR settings: 94°C for 5min, 94°C for 30s, 55°C or 56°C for 30s-1min, 72°C for 1-2min, 72°C-10min for 25-35 cycles using Taq polymerase. PCR products were purified using the Qiagen PCR purification kit or extracted from 1.5-2% agarose gel using the Qiagen gel purification kit. In some cases, a second PCR amplification course was completed to have sufficient PCR product for sub-cloning and sequencing. PCR products were either directly sent for sequencing, or sub-cloned using the TOPO TA cloning kit (vector pCR2.1-TOPO, #49-0030 or pCR4-TOPO, #45-0641; Life Technologies, Waltham, MA) followed by transformation into OneShot TOP10 E.coli using LB-Kan for selection. Plasmids were isolated using the Qiagen miniprep kit. Products were submitted to the Stanford PAN facility or ElimBiopharm (Hayward, CA) for sanger sequencing using the same primers above.

**HLA-ABC and HLA-DR expression Analysis**

To evaluate HLA-ABC and HLA-DR expression, samples (PBMCs from primary AML tumor samples or AML cell line samples) were thawed at 37°C, washed (RPMI 10% FBS), and stained per routine using approximately 5 X 10^5^-1.5 X 10^6^ cells/ml with the following antibodies: CD45-Ax700 (Biolegend, IgG1), HLA-ABC-APC (BD, IgG1), HLA-DR-PerCP-Cy5.5 (BD, IgG2a, clone L243), CD34-PE (BD, IgG1), CD33-FITC (BD, IgG1). Approximately 10,000 to 30,000 events per sample were collected on a BD LSRII. Patient samples and cell lines were analyzed separately due to baseline cell size differences. Flow data were analyzed on Cytobank software (Mountain View, CA) with gating strategy was as follows: singlets using FSH/FSA; live cells using SSA/FSA. The percentage of blasts from PBMC specimens from primary tumor samples was determined using the dim/moderate CD45 versus low SSC-H for typical blast gate and high CD45 versus moderate SSC-H for myelomonocytic blast gate. HLA Class I and II staining were analyzed on the live cell gate. HLA-ABC and HLA-DR median fluorescent intensity (MFI) was compared to the number of distinct Class I or II peptides obtained for each sample using Pearson’s correlation.

**MHC-class I and II immunopeptidome purification**

MHC-class I and II immunopeptidomes were extracted from patient samples (1 X 10^8^ cells per MHC preparation) or cell lines (1 X 10^9^ cells per MHC preparation) as previously described [1–3]. In brief, cells were lysed for 20 min on ice in 20 mM Tris-HCl (pH 8), 150 mM NaCl, 1 % (w/v) CHAPS, 0.2 mM PMSF, 1x Halt™ Protease and Phosphatase Inhibitor Cocktail (Thermo Fisher Scientific, Rockford, USA) supplemented with complete protease inhibitor cocktail (Roche, Mannheim, Germany). The lysate was centrifuged (2x30 min, 13,200 rpm at 4°C) and the resulting supernatant was precleared for 30 min using rProtein A Sepharose fast-flow beads (GE Healthcare, Uppsala, Sweden). Precleared lysate was incubated with either the pan HLA-A-, B-, and C- antibody W6/32 [4] or the HLA-DR specific antibody L243 (produced and purified by Genentech from our hybridoma) [5] coupled to rProtein A Sepharose fast-flow beads for 5h at 4 °C. Following the immune-capture of MHC-class I or II molecules, beads were washed with TBS (pH 7.4) and peptides were eluted from the purified MHC-molecules using 10% acetic acid. The eluate was then passed through a 10 kDa MWCO size filter, followed by a concentration step using vacuum centrifugation, before being desalted on C18 based STAGE tips[6] and stored at -80°C until LC-MS/MS analysis.

**Analysis of HLA Peptides by Mass Spectrometry**

Isolated HLA peptides were reconstituted in 12 µl of 0.1 % formic acid and analyzed on an LTQ Orbitrap Elite mass spectrometer (Thermo Fisher Scientific, Bremen, Germany) or a Fusion Lumos mass spectrometer (Thermo Fisher Scientific, San Jose, USA). Peptides were separated by capillary reverse phase chromatography on 20-24 cm reversed phase columns (100 µm inner diameter, packed in-house with ReproSil-Pur C18-AQ 3.0 m resin (Dr. Maisch GmbH)). The Orbitrap Elite was equipped with an Eksigent ekspert nanoLC-425 system (Sciex, Framingham, USA) using a two-step linear gradient with 4–25 % buffer B (0.1% (v/v) formic acid and 5% DMSO in acetonitrile) for 120 min followed by 25-40 % buffer B for 30 min. All AML patient samples were measured with the Orbitrap Elite system and analyzed in three injections with complementary acquisition methods as previously described [1]. The Fusion Lumos was equipped with a Dionex Ultimate 3000 LC-system and used a similar two-step linear gradient with 4–25 % buffer B (0.1% (v/v) formic acid in acetonitrile) for 80 min followed by 25-45% buffer B for 10 min. Samples analyzed with Fusion Lumos system (OCI-AML3 and MV4-11 cell lines) were acquired in top speed data dependent mode with a duty cycle time of 3 s. Full MS scans were acquired in the Orbitrap mass analyzer with a resolution of 120 000 (FWHM) and m/z scan range of 340-1540. For MHC-Class I samples, precursor ions with mass range of 700-1800 and charge state 1-5 and intensity threshold above 50,000 were selected for fragmentation using higher-energy collisional dissociation (HCD) with quadrupole isolation, isolation window of 1.6 m/z and normalized collision energy of 30%. For MHC-Class II samples, the mass range was set to 700-2760 and charge state 2-6. HCD fragments were analyzed in the Orbitrap mass analyzer with a resolution of 15,000 (FWHM). Fragmented ions were dynamically excluded from further selection for a period of 30 seconds. Each sample was measured twice, once with above described HCD method and a second analysis using a method which toggled HCD and electron transfer dissociation (ETD) fragmentation modes for each isolated precursor using the following parameters for ETD: charge state 2 was excluded, calibrated charge dependent ETD parameters were enabled and 25% of supplemental collision energy was used. The AGC target was set to 400000 and 50000 for full FTMS scans and FTMS2 scans. The maximum injection time was set to 50 ms and 200 ms for full FTMS scans and FTMS2 scans.

**References**

1. Khodadoust MS, Olsson N, Wagar LE, Haabeth OAW, Chen B, Swaminathan K, et al. Antigen presentation profiling reveals recognition of lymphoma immunoglobulin neoantigens. Nature. 2017;543(7647):723–7.

2. Hunt DF, Henderson RA, Shabanowitz J, Sakaguchi K, Michel H, Sevilir N, et al. Characterization of peptides bound to the class I MHC molecule HLA-A2.1 by mass spectrometry. Science (80- ). 1992;255(5049):1261–3.

3. Olsson N, Schultz LM, Zhang L, Khodadoust MS, Narayan R, Czerwinski DK, et al. T-cell immunopeptidomes reveal cell subtype surface markers derived from intracellular proteins. Proteomics. 2018;1700410.

4. Barnstable CJ, Bodmer WF, Brown G, Galfre G, Milstein C, Williams AF, et al. Production of monoclonal antibodies to group A erythrocytes, HLA and other human cell surface antigen-new tools for genetic analysis. Cell. 1978;14(May):9–18.

5. Lampson LA, Levy R. Two populations of Ia-like molecules on a human B cell line. J Immunol. 1980;125(1):293–9.

6. Rappsilber J, Ishihama Y, Mann M. Stop and go extraction tips for matrix-assisted laser desorption/ionization, nanoelectrospray, and LC/MS sample pretreatment in proteomics. Anal Chem. 2003;75(3):663–70.
