# Supplementary material for: Acute myeloid leukemia immunopeptidome reveals HLA presentation of mutated nucleophosmin
Source: PLoS One. 2019 Jul 10;14(7):e0219547. doi: 10.1371/journal.pone.0219547 (PMC6619824; doi:10.1371/journal.pone.0219547)
Supplement: S1 Table — Common recurrent AML mutations of interest with their frequency reported in literature and origination of clinical mutation data annotated for patient samples in study. (DOCX) [file pone.0219547.s009.docx]

**Supplementary Table 1**. Common recurrent AML mutations of interest with their frequency reported in literature and origination of clinical mutation data annotated for patient samples in study.

| GENE | Frequently reported Mutations in literature | Frequency of Gene Mutations in Adult AML Reported in Literature | Origination of clinical mutation data and specific mutations clinically tested for patient samples in this study |
| --- | --- | --- | --- |
| NPM1 | L287, W288 | 27-35%  (45-64% normal karyotype AML) | Stanford Molecular Lab |
| DNMT3A | R882H  R882C | 20% | Stanford SNAPSHOT PANEL  EXON 23: 2644C>T (R882C), 2644C>A (R882S), 2645G>A (R882H), 2645G>C (R882P) |
| IDH1 | R132C  R132H | 7% | Stanford SNAPSHOT PANEL  EXON 4: 394C>T (R132C), 394C>A (R132S), 394C>G (R132G), 395G>A  (R132H), 395G>T (R132L), 395G>C (R132P) |
| IDH2 | R140Q  R172K | 8% | Stanford SNAPSHOT Panel  EXON 4: 419G>A (R140Q), 419G>T (R140L), 514A>G (R172G), 515G>T  (R172M), 515G>A (R172K) |
| KIT | D816V  D816Y  Y418S | 4-6% | Stanford Molecular Lab  Exon 8 and Exon 17 mutations |
| FLT3 | FLT-ITD(indels)  FLT3-TKD:  D835Y  D835E  D835H | FLT3-ITD: 20%  FLT3-TKD: 5-10% | Stanford Molecular Lab or LabPMM |
| NRAS/KRAS | G12C  G12D  G12V  G13D  Q61H  Q61K  Q61P  Q61R | NRAS: 10%  KRAS: 2% | Stanford SNAPSHOT PANEL  KRAS: EXON 2: 34G>C (G12R), 34G>A (G12S), 34G>T (G12C), 35G>C (G12A),  35G>A (G12D), 35G>T (G12V), 37G>C (G13A), 37G>A (G13S), 37G>T (G13C), 38G>C (G13A), 38G>A (G13D), 38G>T (G13V)  NRAS: EXON 2: 34G>T (G12C), 34G>A (G12S), 34G>C (G12R), 35G>T (G12C),  35G>A (G12D), 35G>C (G12A), 37G>T (G13C), 37G>C (G13R), 37G>A (G13S), 38G>T (G13V), 38G>C (G13A), 38G>A (G13D), 181C>A (Q61K), 181C>G (Q61E), 183 A>T (Q61H), 183A>G (Q61Q), 183A>C (Q61H) |
