# Supplementary material for: Acute myeloid leukemia immunopeptidome reveals HLA presentation of mutated nucleophosmin
Source: PLoS One. 2019 Jul 10;14(7):e0219547. doi: 10.1371/journal.pone.0219547 (PMC6619824; doi:10.1371/journal.pone.0219547)
Supplement: S2 Table — (A) List of eluted HLA Class I and Class II peptides from patient samples (n = 13) per source proteins of previously published leukemia associated antigens. The number of distinct Class I or II peptides in the combined data set derived from patient samples were counted. (B) List of eluted HLA Class I and Class II peptides from tumor cell lines (n = 2) per source proteins of previously published leukemia associated antigens. The number of distinct Class I or II peptides in the combined data set derived from the two cell lines were counted. (DOCX) [file pone.0219547.s010.docx]

**S2 Table.**

**Supplementary Table 2A**. List of eluted HLA Class I and Class II peptides from patient samples (n=13) from source proteins of previously published leukemia associated antigens. The number of distinct Class I or II peptides in the combined data set derived from patient samples were counted.

| **Leukemia Associated Antigen: Protein Name** | **Uniprot ID** | **Peptide Sequences** | **Class I or Class II** | **Sample** | **Overall Distinct Class I** | **Overall Distinct Class II** |
| --- | --- | --- | --- | --- | --- | --- |
| AURKA | O14965 | None | NA | NA | 0 | 0 |
| BCL2 | P10415 | None | NA | NA | 0 | 0 |
| Bl1 | P55061 | None | NA | NA | 0 | 0 |
| BIRC5/IAP4 | O15392 | None | NA | NA | 0 | 0 |
| BMI1 | P35226 | None | NA | NA | 0 | 0 |
| BRAP | Q7Z569 | None | NA | NA | 0 | 0 |
| CA9/G250 | Q16790 | None | NA | NA | 0 | 0 |
| CCNA1 | P78396 | SLSEIVPC[cys]L | Class I | AML009 | 1 | 0 |
| CCNB1 | P14635 | None | NA | NA | 0 | 0 |
| CCNE1 | P24864 | None | NA | NA | 0 | 0 |
| CL12A/CLL1 | Q5QGZ9 | EELQRNISL  EELQRNISL  NPVQLGSTY  VDNIINSSAWVIR | Class I  Class I  Class I  Class 2 | AML001  AML013  AML015  AML013 | 2 | 1 |
| CYP1B1 | Q16678 | ERAIHQAL | Class I | AML013 | 1 | 0 |
| EXOS5/CML28 | Q9NQT4 | HPRTSITVVL | Class I | AML003 | 1 | 0 |
| HOXA9 | P31269 | DAADELSVGRY  DELSVGRY  KATVFGASW  KATVFGASW | ClassI  ClassI  ClassI  ClassI | AML008  AML006  AML009  AML016 | 3 | 0 |
| KLF2 | Q9Y5W3 | None | NA | NA | 0 | 0 |
| MAGE/MAGEA1 | P43355 | None | NA | NA | 0 | 0 |
| MAGED1 | Q9Y5V3 | DVYPEIIER  RPKSAFKVQNA  RPKSAFKVQNA | Class I  Class I  Class I | AML015  AML008  AML016 | 2 | 0 |
| MCL1 | Q07820 | ESITDVLVR  ETAFQGMLR  ETAFQGMLR  GRIVTLISF  GRIVTLISF  HVFSDGVTNW  SRKALETLR | Class I  Class I  Class I  Class I  Class I  Class I  Class I | AML015  AML011  AML015  AML009  AML015  AML006  AML015 | 5 | 0 |
| MPO | P05164 | AVSNEIVRFPTDQLTPD  AVSNEIVRFPTDQLTPD  AVSNEIVRFPTDQLTPDQ  DFTPEPAAR  DFTPEPAARAS  DFTPEPAARASF  DFTPEPAARASFV  DGERLYQEARKIVG  DGERLYQEARKIVG  DGERLYQEARKIVG  DGERLYQEARKIVGA  DGERLYQEARKIVGAM  DGERLYQEARKIVGAM[ox]  DGERLYQEARKIVGAM[ox]V  DHDLDFTPEPAARAS  DHDLDFTPEPAARASF  DHDLDFTPEPAARASFV  DHDLDFTPEPAARASFVT  DHDLDFTPEPAARASFVTG  DLDFTPEPAARAS  DLDFTPEPAARASFV  DNRYQPMEPNPRVP  DNRYQPMEPNPRVPL  DNTGITTVSKNNI  DNTGITTVSKNNIF  DNTGITTVSKNNIF  DNTGITTVSKNNIFM[ox]  DNTGITTVSKNNIFM[ox]  ERLYQEARKIVG  ETVGQLGTVLR  FMQWGQLLDHDLD  GERLYQEARKIVG  GLLAVNQRFQDNGRA  GVPFFSSLR  HDLDFTPEPAARA  HDLDFTPEPAARAS  HDLDFTPEPAARASF  HDLDFTPEPAARASFV  HDLDFTPEPAARASFVT  LDFTPEPAARAS  LDFTPEPAARASFV  LDHDLDFTPEPAARAS  LDHDLDFTPEPAARASFV  LDHDLDFTPEPAARASFVT  LDHDLDFTPEPAARASFVTG  LDNRYQPM[ox]EPNPRVPL  LDNRYQPMEPNPRVP  LDNRYQPMEPNPRVPL  LDNRYQPMEPNPRVPL  LGLLAVNQRFQDN  LGLLAVNQRFQDNG  LGLLAVNQRFQDNGR  LGLLAVNQRFQDNGR  LGLLAVNQRFQDNGRA  MQWGQLLDHDLDFTPEPAAR  MVYGSEEPLAR  MVYGSEEPLAR  NGFPVALAR  NQLGLLAVNQRFQDNG  NQLGLLAVNQRFQDNGR  NQLGLLAVNQRFQDNGRA  NQLGLLAVNQRFQDNGRAL  NTGITTVSKNNIFM[ox]  QLGLLAVNQRFQDNGR  QLGLLAVNQRFQDNGRA  RAVSNEIVRFPTDQLTPDQ  RLYQEARKIVG  SGSASPMELLSYFKQPVAATR  SNEIVRFPTDQLTPD  SNEIVRFPTDQLTPD  SNEIVRFPTDQLTPD  SNEIVRFPTDQLTPD  SNEIVRFPTDQLTPDQ  SNEIVRFPTDQLTPDQ  SNQLGLLAVNQRFQDNGRA  VLRNLKLARKLM[ox]EQYG  VSNEIVRFPTDQ  VSNEIVRFPTDQLTPD  VSNEIVRFPTDQLTPD  VSNEIVRFPTDQLTPD  VSNEIVRFPTDQLTPD  WDGERLYQEARKIVG | Class 2  Class 2  Class 2  Class I  Class 2  Class 2  Class 2  Class I  Class 2  Class 2  Class 2  Class 2  Class 2  Class 2  Class 2  Class 2  Class 2  Class 2  Class 2  Class 2  Class 2  Class 2  Class 2  Class 2  Class 2  Class 2  Class 2  Class 2  Class 2  Class I  Class 2  Class 2  Class 2  Class I  Class 2  Class 2  Class 2  Class 2  Class 2  Class 2  Class 2  Class 2  Class 2  Class 2  Class 2  Class 2  Class 2  Class 2  Class 2  Class 2  Class 2  Class I  Class 2  Class 2  Class 2  Class I  Class I  Class I  Class 2  Class 2  Class 2  Class 2  Class 2  Class 2  Class 2  Class 2  Class 2  Class 2  Class 2  Class 2  Class 2  Class 2  Class 2  Class 2  Class 2  Class 2  Class 2  Class 2  Class 2  Class 2  Class 2  Class 2 | AML006  AML011  AML011  AML011  AML015  AML015  AML015  AML015  AML010  AML015  AML015  AML010  AML010  AML010  AML015  AML015  AML015  AML015  AML015  AML015  AML015  AML010  AML010  AML011  AML009  AML011  AML009  AML011  AML015  AML015  AML011  AML015  AML011  AML015  AML015  AML015  AML015  AML015  AML015  AML015  AML015  AML015  AML015  AML015  AML015  AML010  AML010  AML009  AML010  AML011  AML011  AML011  AML011  AML011  AML015  AML011  AML015  AML011  AML011  AML011  AML011  AML011  AML011  AML011  AML011  AML006  AML015  AML006  AML003  AML006  AML009  AML011  AML006  AML011  AML011  AML015  AML003  AML003  AML006  AML009  AML011  AML015 | 7 | 62 |
| MPP11 | Q99543 | EVFTPVFER  EVFTPVFER | Class I  Class I | AML001  AML015 | 1 | 0 |
| MSLN | Q13421 | None | NA | NA | 0 | 0 |
| MUC1 | P15941 | None | NA | NA | 0 | 0 |
| NUDCD1/CML66 | Q96RS6 | None | NA | NA | 0 | 0 |
| NUP214/NU214 | P35658 | RPVAPSGTAL  VSNKYGLVF | Class I  Class I | AML003  AML014 | 2 | 0 |
| NUSAP1 | Q9BXS6 | ESIDQYIER  SVASTPISQR | Class I  Class I | AML015  AML015 | 2 | 0 |
| P2X5/P2RX5 | Q93086 | None | NA | NA | 0 | 0 |
| PASD1 | Q8IV76 | None | NA | NA | 0 | 0 |
| PRAME | P78395 | None | NA | NA | 0 | 0 |
| PRTN3/PR3 | P24158 | EIVGGHEAQPHSR  FLNNYDAENKLNDVL  IHPSFVLTAAHC[cys]LRDIPQ  LNNYDAENKLND  LNNYDAENKLNDV  LNNYDAENKLNDVL  LNNYDAENKLNDVLL  YVDWIRSTLR  YVDWIRSTLR  YVDWIRSTLRR | Class I  Class 2  Class 2  Class 2  Class 2  Class 2  Class 2  Class I  Class 2  Class I | AML015  AML015  AML015  AML015  AML015  AML015  AML015  AML010  AML010  AML010 | 3 | 7 |
| RAGE1/MOK | Q9UQ07 | None | NA | NA | 0 | 0 |
| RGS5 | O15539 | None | NA | NA | 0 | 0 |
| RHAMM/HMMR | O75330 | ELTEEINKW | Class I | AML006 | 1 | 0 |
| RUNX1/AML1 | Q01196 | ARFNDLRFVGR  ARFNDLRFVGR  ASLNHSTAFNPQPQ  ASLNHSTAFNPQPQ  ASLNHSTAFNPQPQ  DVPDGTLVTVM  ERSPPRIL  ITVDGPREPR  NQVARFNDL  RFNDLRFVGR  RFNDLRFVGR  SLNHSTAFNPQPQ  SLNHSTAFNPQPQ  TPIS[p]PGRASGM | Class I  Class I  Class I  Class I  Class I  Class I  Class I  Class I  Class I  Class I  Class I  Class I  Class I  Class I | AML009  AML015  AML008  AML009  AML011  AML008  AML008  AML011  AML013  AML006  AML014  AML006  AML008  AML015 | 9 | 0 |
| SSX2 | Q16385 | None | NA | NA | 0 | 0 |
| SSX2IP | Q9Y2D8 | KAFEEERASW | Class I | AML010 | 1 | 0 |
| TERT | O14746 | None | NA | NA | 0 | 0 |
| WT1 | P19544 | None | NA | NA | 0 | 0 |

**Supplementary Table 2B.** List of eluted HLA Class I and Class II peptides from tumor cell lines (n=2) from source proteins of previously published leukemia associated antigens. The number of distinct Class I or II peptides in the combined data set derived from the two cell lines were counted.

| **Leukemia Associated Antigen: Protein Name** | **Uniprot ID** | **Peptides** | **Class I or Class II** | **Cell Line** | **Overall Distinct Class I** | **Overall Distinct Class II** |
| --- | --- | --- | --- | --- | --- | --- |
| AURKA | O14965 | GELKIADFGW  KIADFGWSV | Class I  Class I | OCIAML3  OCIAML3 | 2 | 0 |
| BCL2 | P10415 | None | NA | NA | 0 | 0 |
| Bl1 | P55061 | None | NA | NA | 0 | 0 |
| BIRC5/IAP4 | O15392 | None | NA | NA | 0 | 0 |
| BMI1 | P35226 | TLQDIVYKL | Class I | OCIAML3 | 1 | 0 |
| BRAP | Q7Z569 | EEINNMKTKF  QEQLRDVMFY  YWENKIVRI | Class I | OCIAML3 | 3 | 0 |
| CA9/G250 | Q16790 | None | NA | NA | 0 | 0 |
| CCNA1 | P78396 | FVYITDDTYTK  C[cys]LANYTVNK  DRFLSC[cys]MSV  LEADPFLKY | Class I  Class I  Class I  Class I | MV411  MV411  MV411  MV411 | 4 | 0 |
| CCNB1 | P14635 | AENKAKINM  TVSIIDRF  VMVNQGLTKHMTV  VVMVNQGLTK  NTYTKHQIR  EEQAVRPKY  PVPEPEPEPEPEPVKE  DRFMQNNC[cys]V  EEEQAVRPKY  AEEDLC[cys]QAF  MTVSIIDRF | Class I  Class I  Class I  Class I  Class I  Class I  Class 2  Class I  Class I  Class I  Class I | OCIAML3  OCIAML3  OCIAML3  MV411  MV411  OCIAML3  OCIAML3  MV411  OCIAML3  OCIAML3  MV411 | 10 | 1 |
| CCNE1 | P24864 | IYPPKLHQF  KLHRETFYL  EEIYPPKLHQF  HEVLLPQY  HFLEQHPLL  ALYHFSSSELMQK | Class I  Class I  Class I  Class I  Class I  Class I | OCIAML3  OCIAML3  OCIAML3  MV411  OCIAML3  MV411 | 6 | 0 |
| CL12A/CLL1 | Q5QGZ9 | None | NA | NA | 0 | 0 |
| CYP1B1 | Q16678 | ERAIHQAL  DQVVGRDRL  NGERAIHQAL | Class I  Class I  Class I | MV411  MV411  MV411 | 3 | 0 |
| EXOS5/CML28 | Q9NQT4 | AQAASQHVF | Class I | OCIAML3 | 1 | 0 |
| HOXA9 | P31269 | DAADELSVGR  DELSVGRY | Class I  Class I | MV411  MV411 | 2 | 0 |
| KLF2 | Q9Y5W3 | None | NA | NA | 0 | 0 |
| MAGE/MAGEA1 | P43355 | None | NA | NA | 0 | 0 |
| MAGED1 | Q9Y5V3 | KEIDKEEHLY  EYTDVYPEI  DVYPEIIER | Class I  Class I  Class I | OCIAML3  OCIAML3  MV411 | 3 | 0 |
| MCL1 | Q07820 | SLSRVMIHV  ESITDVLVR  ESITDVLVRTK  EVPDVTATPARL  DELYRQSL  ETAFQGMLR  ETLRRVGDGVQR  ISFGAFVAK | Class I  Class I  Class I  Class I  Class I  Class I  Class I  Class I | OCIAML3  MV411  MV411  MV411  MV411  MV411  MV411  MV411 | 8 | 0 |
| MPO | P05164 | None | NA | NA | 0 | 0 |
| MPP11 | Q99543 | KMMEEVEKL | Class I | OCIAML3 | 1 | 0 |
| MSLN | Q13421 | None | NA | NA | 0 | 0 |
| MUC1 | P15941 | None | NA | NA | 0 | 0 |
| **NUDCD1/**CML66 | Q96RS6 | AEVKLRDDQY  RIMNLTVML  EESSSLCRF | Class I  Class I  Class I | OCIAML3  OCIAML3  OCIAML3 | 3 | 0 |
| NUP214/NU214 | P35658 | FTAPPVLGK  SEYGSIIAF  SVFGSGNTGR | Class I  Class I  Class I | MV411  MV411  MV411 | 3 | 0 |
| NUSAP1 | Q9BXS6 | SEIKISNPTEF  YLNQHVNRI  HVTVSGGTPK  AVLGTHKLK  EEHNSMNEL  HFEEHNSM  HEAHFKEM  SVASTPISQR  SVASTPISQRR  ESIDQYIER  ESIDQYIERK  VASTPISQR  ASTPISQR | Class I  Class I  Class I  Class I  Class I  Class I  Class I  Class I  Class I  Class I  Class I  Class I  Class I | OCIAML3  OCIAML3  MV411  MV411  OCIAML3  OCIAML3  MV411  MV411  MV411  MV411  MV411  MV411  MV411 | 13 | 0 |
| P2X5/P2RX5 | Q93086 | YQDVDTSL  IFIKNHIRF | Class I  Class I | OCIAML3  OCIAML3 | 2 | 0 |
| PASD1 | Q8IV76 | None | NA | NA | 0 | 0 |
| PRAME | P78395 | None | NA | NA | 0 | 0 |
| PRTN3/PR3 | P24158 | AEIVGGHEAQPH  DAENKLNDVLL  DWIRSTLRR  WIRSTLRR  YVDWIRSTLRR  EIVGGHEAQPHSR  EIVGGHEAQPHSR  IVGGHEAQPHSRPYM  IVGGHEAQPHSRPYMA  VGGHEAQPHSR  IHPSFVLTAAHC[119.00]LR  IHPSFVLTAAHC[119.00]LRD  YVDWIRST  YVDWIRSTLR  VVLGAHNVRTQEPTQQHFSVAQVF  GGHEAQPHSR | Class 2  Class 2  Class 2  Class 2  Class 2  Class I  Class 2  Class 2  Class 2  Class I  Class 2  Class 2  Class 2  Class 2  Class 2  Class I | MV411  MV411  MV411  MV411  MV411  MV411  MV411  MV411  MV411  MV411  MV411  MV411  MV411  MV411  MV411  MV411 | 3 | 13 |
| RAGE1/MOK | Q9UQ07 | None | NA | NA | 0 | 0 |
| RGS5 | O15539 | None | NA | NA | 0 | 0 |
| RHAMM/HMMR | O75330 | KILEKEIRV  KNDEILSL  RLNAALREK  TEIKEITVSF  KLLGHQNLK  QEQLNKIRDSY  KLLEYIEEI  DTTLPASAR  EYIEEISC  AEDVQHQIL  TTAELTEEINK  AELTEEINKW  KLQEELNKV | Class I  Class I  Class I  Class I  Class I  Class I  Class I  Class I  Class I  Class I  Class I  Class I  Class I | OCIAML3  MV411  MV411  MV411  MV411  OCIAML3  OCIAML3  MV411  OCIAML3  OCIAML3  MV411  OCIAML3  OCIAML3 | 13 | 0 |
| RUNX1/AML1 | Q01196 | ITVDGPREPR  TVDGPREPR  TVFTNPPQV  NQVARFNDL  EEAVWRPY  IPVDASTSR  LEEAVWRPY | Class I  Class I  Class I  Class I  Class I  Class I  Class I | MV411  MV411  OCIAML3  MV411  MV411  MV411  MV411 | 7 | 0 |
| SSX2 | Q16385 | None | NA | NA | 0 | 0 |
| SSX2IP | Q9Y2D8 | None | NA | NA | 0 | 0 |
| TERT | O14746 | None | NA | NA | 0 | 0 |
| WT1 | P19544 | None | NA | NA | 0 | 0 |
