# Supplementary material for: Acute myeloid leukemia immunopeptidome reveals HLA presentation of mutated nucleophosmin
Source: PLoS One. 2019 Jul 10;14(7):e0219547. doi: 10.1371/journal.pone.0219547 (PMC6619824; doi:10.1371/journal.pone.0219547)
Supplement: S3 Table — Comparison of use of FDR 1% versus <11% for analysis of Class I (A) and Class II (B) eluted peptides for to identify peptides from recurrent mutations from patient samples and cell lines. (DOCX) [file pone.0219547.s011.docx]

**Supplemental Table 3**. Peptides from recurrent mutations. Comparison of use of FDR 1% versus <11% for analysis of Class I (A) and Class II (B) eluted peptides for to identify peptides from recurrent mutations from patient samples and cell lines.

**Suppl. Table 3A**

| **Sample** | **Protein** | **1 % FDR cutoff**  **HLA Class I (PEP-score, FDR)** | **<11 % FDR cutoff**  **HLA Class I (PEP-score, FDR)** |
| --- | --- | --- | --- |
| AML003 | Mutated NPM1 | AVEEVSLRK (0.0001316, 0) | AVEEVSLRK (0.000132, 0) |
| AML006 | Mutated NPM1 | NA | AVEEVSLRK (0.3398, 8.2) |
| OCI-AML3 | Mutated NPM1 | NA | C[cys]LAVEEVSL (0.4799, 10.67) |

**Suppl. Table 3B**

| **Sample** | **Protein** | **1 % FDR cutoff**  **HLA Class II (PEP-score, FDR %)** | **<11 % FDR cutoff**  **HLA Class II (PEP-score, FDR %)** |
| --- | --- | --- | --- |
| AML003 | Mutated NPM1 | AVEEVSLRK (0.003796, 0) | AVEEVSLRK (0.003796, 0) |
| AML006 | Mutated NPM1 | AVEEVSLRK (1.65x10^-7^, 0) | AVEEVSLRK (1.65x10^-7^, 0) |
| OCI-AML3 | Mutated NPM1 | AVEEVSLRK (0.000003586, 0) | AVEEVSLRK (0.000003586, 0) |
| OCI-AML3 | Mutated NPM1 | LAVEEVSLRK (0.000002, 0) | LAVEEVSLRK (0.000002, 0) |
| OCI-AML3 | Mutated NPM1 | VEEVSLRK (0.01108, 0.1) | VEEVSLRK (0.01108, 0.1) |
| OCI-AML3 | Mutated NPM1 | AVEEVSLR (0.000125, 0) | AVEEVSLR (0.000125, 0) |
| AML016 | FLT3_D835E | NA | FGLAREIMSDSN[deam]Y ( 0.3888, 4.8) |
